# A novel stabilization mechanism for the type VI secretion system sheath

**DOI:** 10.1073/pnas.2008500118

**Published:** 2021-02-08

**Authors:** Patricia Bernal, R. Christopher D. Furniss, Selina Fecht, Rhoda C. Y. Leung, Livia Spiga, Despoina A. I. Mavridou, Alain Filloux

**Affiliations:** ^a^MRC Centre for Molecular Bacteriology and Infection, Department of Life Sciences, Imperial College London, SW7 2AZ London, United Kingdom;; ^b^Department of Biology, Faculty of Sciences, Universidad Autónoma de Madrid, 28049 Madrid, Spain;; ^c^Departamento de Microbiología, Facultad de Biología, Universidad de Sevilla, 41012 Seville, Spain;; ^d^Department of Molecular Biosciences, University of Texas at Austin, Austin, TX 78712

**Keywords:** type VI secretion system, TssA, TagB, sheath stabilization, *Pseudomonas*

## Abstract

The type VI secretion system (T6SS) is a phage-derived contractile nanomachine primarily involved in interbacterial competition. Its pivotal component, TssA, is indispensable for the assembly of the T6SS sheath structure, the contraction of which propels a payload of effector proteins into neighboring cells. Despite their key function, TssA proteins exhibit unexpected diversity and exist in two major forms, a short form (TssA_S_) and a long form (TssA_L_). While TssA_L_ proteins interact with a partner, called TagA, to anchor the distal end of the extended sheath, the mechanism for the stabilization of TssA_S_-containing T6SSs remains unknown. Here we discover a class of structural components that interact with short TssA proteins and contribute to T6SS assembly by stabilizing the polymerizing sheath from the baseplate. We demonstrate that the presence of these components is important for full sheath extension and optimal firing. Moreover, we show that the pairing of each form of TssA with a different class of sheath stabilization proteins results in T6SS apparatuses that either reside in the cell for some time or fire immediately after sheath extension. We propose that this diversity in firing dynamics could contribute to the specialization of the T6SS to suit bacterial lifestyles in diverse environmental niches.

## Significance

The T6SS is a microscopic harpoon that bacteria use to deliver toxins into neighboring cells. While its complex assembly process has been extensively studied, it remains unclear how the two forms (long and short) of the pivotal TssA protein affect T6SS function. TssA promotes baseplate formation, orchestrates sheath extension and, in its long form, interacts with a partner protein to anchor the extending sheath at the opposing side of the cell for up to 10 min. Here we demonstrate that short TssA proteins assist sheath stabilization by associating with a yet undescribed class of T6SS proteins that accumulate at the baseplate. These T6SSs fire in seconds; therefore, this discovery provides insight into the mechanism underpinning the different fighting strategies observed across T6SS-carrying bacteria.

Bacteria live in complex polymicrobial communities that are shaped by interspecies cooperation and competition. As resources are limited, antagonistic strategies are a major driver of survival and success for bacterial populations. One of the most elaborate bacterial weapons is the type VI secretion system (T6SS), which not only promotes interbacterial and interkingdom competition (1–3), but also is involved in the interaction of bacteria with their hosts (4, 5) and the acquisition of both nutrients (6, 7) and genetic material (8, 9).

**Fig. 1.**
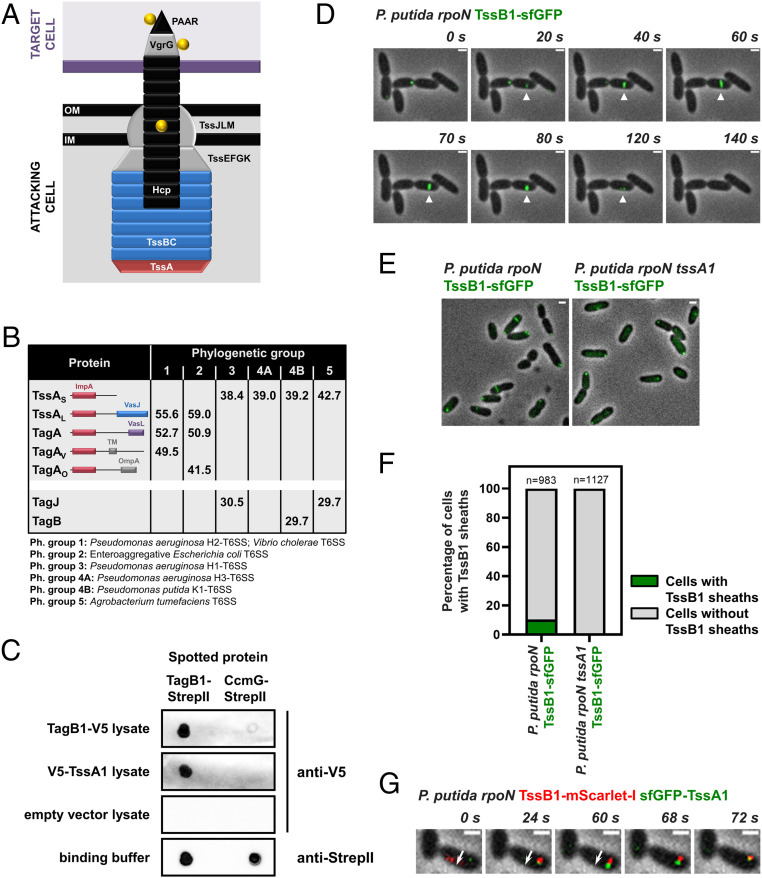
Short TssA proteins interact with a yet undescribed class of T6SS structural components. (*A*) Schematic representation of a T6SS apparatus at the moment of firing. The Hcp tube (black), the VgrG/PAAR tip complex (gray/black), and their associated effectors (yellow) are propelled from the attacking cell into a neighboring target cell (purple). The membrane complex and baseplate structures are shown in gray, while the sheath and the cap protein TssA are depicted in blue and red, respectively. (*B*) Synopsis of the results from the in silico analysis presented in Dataset S1. Average protein sizes in kDa are given for T6SS components from each phylogenetic group, and the domain architecture of TssA-like proteins is shown; the N terminus of all TssA-like proteins forms an ImpA domain, while their C terminus varies (28, 29). “TagA” refers to homologs of the *E. coli* TagA anchor protein (30), “TagA_V_” has been described as the anchor for the T6SS of *V. cholerae* (29), and “TagA_O_”, which has a different C terminus than the other two forms of TagA, has not been described previously. Representative examples of well-studied T6SSs from each phylogenetic group are given in the footnote. (*C*) Dot blot assays showing interaction of *P. putida* TagB1 with itself and its cognate TssA1. CcmG-StrepII was used as a binding control protein; similar amounts of pure TagB1-StrepII and CcmG-StrepII were spotted on the membrane. (*D*–*G*) *P. putida* K1-T6SS sheaths display characteristic T6SS behaviors. (*D*) In vivo imaging of *P. putida rpoN* TssB1-sfGFP showing the full cycle of T6SS assembly over ∼120 s for a representative sheath (white arrowhead). The panels are selected images from a fluorescence microscopy time-lapse recording of *P. putida rpoN* expressing TssB1-sfGFP from the native *tssB1* locus (Movie S1); images were recorded every 2 s. (*E*) Formation of *P. putida* sheaths is dependent on TssA1. Shown are representative fluorescence microscopy images of *P. putida rpoN* and *P. putida rpoN tssA1* expressing TssB1-sfGFP from the native *tssB1* locus. (*F*) Quantification of images in *E*. Approximately 10% of cells form sheaths in *P. putida rpoN*, while no sheaths are detected in the isogenic *tssA1* mutant. *n* indicates the number of cells included in the analysis. (*G*) *P. putida* TssA1 localizes to the site of sheath initiation and subsequently migrates at the end of the polymerizing sheath. The white arrow indicates the direction of sheath extension. The panels are selected images from a fluorescence microscopy time-lapse recording of *P. putida rpoN* expressing sfGFP-TssA1 and TssB1-mScarlet-I from the native gene loci; images were recorded every 2 s. (Scale bars in *D*, *E*, and *G*: 1 μm.)

The T6SS apparatus is a contractile nanomachine that delivers proteinaceous effectors to neighboring cells in a contact-dependent manner (10, 11). The system is a multiprotein complex (12) that when fully assembled extends across the entire width of the cell (13). Three main components make up this structure: the membrane complex, the baseplate, and the tail, comprising the contractile sheath and the inner tube. The membrane complex (TssJLM) spans the cell envelope (14–16), providing a platform onto which the baseplate (TssEFGK) docks (17, 18). Once the baseplate is in position, it promotes the polymerization of the contractile sheath (TssBC), which encompasses the Hcp tube topped by the VgrG/PAAR complex (19–21). On sheath contraction (22) the Hcp tube and the VgrG tip, along with their associated effectors, are propelled outside the attacker and into neighboring cells (10, 11). This is followed by ClpV-dependent sheath disassembly and recycling before another round of firing (23–25). A schematic representation of a T6SS at the moment of sheath contraction is shown in Fig. 1*A*.

TssA is a core structural component essential for T6SS function (26, 27). TssA promotes priming and polymerization of the sheath, whereby after recruiting the remaining baseplate proteins, it is displaced by the growing sheath and migrates at its distal end, eventually reaching the other side of the cell (27). Because of its central role, TssA is found in all T6SSs; nonetheless, it is not very well conserved. Notably, TssA exists in two major forms, a long 55- to 60- kDa protein (TssA_L_) and a shorter ∼40 kDa version (TssA_S_) (28, 29) (Fig. 1*B*). The structure and function of prototypical TssA_L_ proteins have been extensively studied (27–29), and the *Escherichia coli*, *Vibrio cholerae*, and *Aeromonas hydrophila* TssA_L_ are known to form a ring-like structure, where the C terminus of the protein largely occupies the center of the ring (27–29). It has been recently shown that these long TssA forms interact with an accessory TssA-like protein, called TagA, which secures the distal end of the sheath at the opposing cell membrane. This ensures optimal sheath polymerization and allows the sheath to be maintained in its extended conformation for long periods (29, 30). In contrast, *Pseudomonas aeruginosa* and *Burkholderia cenocepacia* TssA_S_, while still ring-like, display structures in which the center of the ring is empty (26, 28). In this case, TagA is not present, and it is unknown how the sheath is stabilized and anchored. To understand the implications of TssA diversity for T6SS assembly dynamics and function, it is necessary to elucidate the relationship among short TssA proteins, the baseplate, and the extending sheath and to determine how TssA_S_-containing T6SSs are stabilized.

## Results

### Short TssA Proteins Interact with a Yet Undescribed Class of T6SS Structural Components.

To assess the core components of T6SSs across different bacterial species, we performed an in silico analysis of 100 T6SS clusters (Dataset S1). We included 20 clusters from each of the five T6SS groups that have been previously identified through phylogenetic analysis of several core T6SS components (31). We found that TssA_L_ proteins dominate phylogenetic groups 1 and 2 and usually co-occur with TagA-like components (Fig. 1*B*; only one TagA protein and its cognate TssA partner are encoded in each cluster). Notably, examples of T6SS clusters that belong to phylogenetic groups 1 and 2 and encode a TssA_L_ without containing a *tagA* gene also exist; for example, the H2-T6SS of *P. aeruginosa* in phylogenetic group 1 (29). On the other hand, short TssA proteins, present in phylogenetic groups 3 to 5, do not co-occur with TagA-like partners but instead are often found to coexist with 30-kDa proteins of unknown function (Fig. 1*B*). As with TssA_L_, there are TssA_S_-encoding clusters that do not contain any of these uncharacterized genes; for example, all clusters from phylogenetic subgroup 4A and some members of phylogenetic group 5. Closer examination of the sequences of short TssA proteins, and more specifically of their C-terminal domain, which is considered less conserved (28), did not reveal any major differences between TssA_S_ proteins that co-occur and those that do not co-occur with these smaller T6SS components (*SI Appendix*, Fig. S2). Nonetheless, phylogenetic analysis showed that TssA_S_ proteins encoded in T6SS operons containing this yet undescribed class of genes often cluster together (*SI Appendix*, Fig. S3).

This observation, along with the fact that in each phylogenetic group, the genes encoding the 30-kDa proteins are always found among other core T6SS genes, prompted us to probe their function. We chose *Pseudomonas putida* as a model organism because we have previously shown that in this bacterium, a representative of these uncharacterized proteins, is encoded by the first gene of the K1-T6SS cluster (32) (*SI Appendix*, Fig. S1), a member of phylogenetic group 4B that also encodes a short TssA protein (Fig. 1*B* and Dataset S1). The gene of interest (PP_5562 or PP3100.1), previously known as *tagX1* (32), is termed *tagB1* (for type VI accessory gene B1) hereinafter.

TagB1 is a predicted cytoplasmic protein of unknown structure. To identify its T6SS interaction partners, we introduced a twin StrepII tag at the C terminus of the native copy of TagB1 and purified the protein in conditions where the K1-T6SS is active using an *rpoN* mutant of *P. putida* KT2440 with increased K1-T6SS expression (32). We identified and quantified the eluted proteins by mass spectrometry and found that TssA1 was the only protein significantly enriched in samples containing TagB1-(StrepII)_2_ compared to untagged control samples (Dataset S2). We validated our mass spectrometry results obtained from the native T6SS apparatus expressed in *P. putida* by dot blot assays. We observed that pure TagB1-StrepII interacted with V5-TssA1 from crude cell extracts, and at the same time we detected a clear interaction of TagB1 with itself (Fig. 1*C*). These associations are specific to TagB1 and TssA1, as no interactions were detected when using a binding control protein or lysates from cells harboring the empty vector (Fig. 1*C*). The fact that TagB1 self-associates, and thus is likely functional in a multimeric state, while also interacting with the core component TssA1 suggests that it is a structural T6SS protein.

### *P. putida* K1-T6SS Sheaths Display Characteristic T6SS Behaviors.

To interrogate whether TagB1 plays a role in assembly of the K1-T6SS, we visualized the apparatus in vivo. The K1-T6SS has been previously shown to be active (32), but it has not been previously imaged by fluorescence microscopy. We generated a *P. putida rpoN* strain that expresses TssB1 fused to superfolder GFP (sfGFP) from the native *tssB1* locus. Using this strain, we detected sheaths in ∼10% of cells at any given time (Fig. 1*F*), and we could visualize the full cycle of T6SS assembly, including sheath initiation, full extension, contraction, and disassembly, over ∼120 s (Fig. 1*D* and Movie S1). Sheath structures with identical dynamics could also be seen in wild-type *P. putida* expressing TssB1-sfGFP (*SI Appendix*, Fig. S4*A*), although they occurred at a lower frequency.

No sheaths were detected in the absence of TssA1 (Fig. 1 *E* and *F*), consistent with the fact that *P. putida tssA1* has been reported to be essential for K1-T6SS function (32) and that in other organisms, sheath formation is observed in vivo only when TssA is present (27). As it has been demonstrated that long TssA proteins from *E. coli*, *V. cholerae*, and *P. aeruginosa* (H2-T6SS) initially localize at the baseplate and subsequently migrate at the tip of the growing sheath (27, 29), we investigated whether a short TssA protein, like *P. putida* TssA1, exhibits similar behavior. To monitor its localization during sheath assembly, we generated a *P. putida rpoN* strain expressing sfGFP-TssA1 and TssB1-mScarlet-I from their native loci. TssA1 localized to the site of sheath initiation, before the sheath structure could be detected, and was then displaced to the distal end of the polymerizing sheath, reaching the other side of the cell before sheath contraction (Fig. 1*G*). This observation for a short TssA protein suggests that despite its two distinct forms, the localization of TssA during T6SS assembly and its interplay with the baseplate and sheath proteins is likely conserved.

### TagB1 Stabilizes Sheath Polymerization from the Baseplate.

**Fig. 2.**
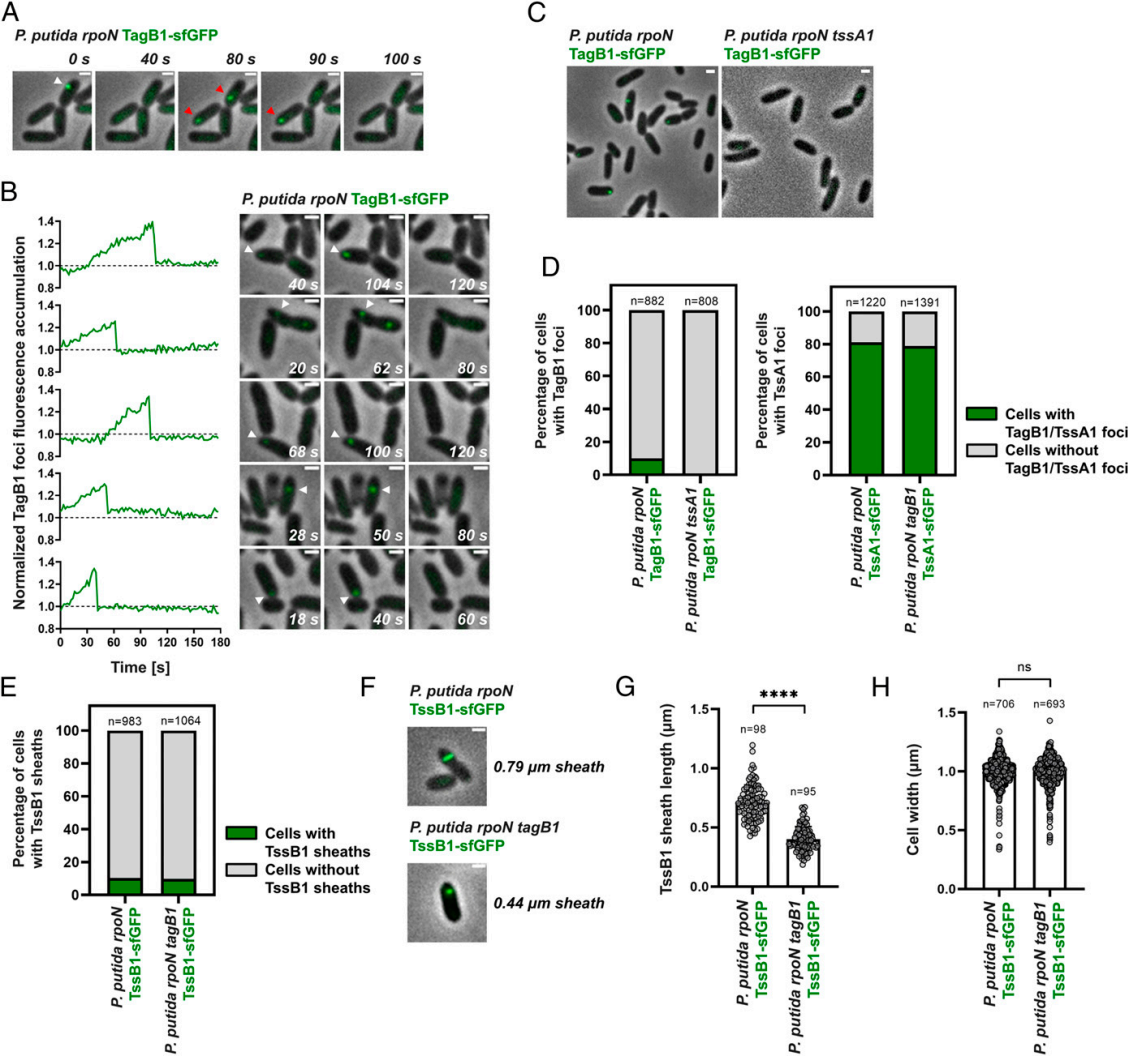
*P. putida* TagB1 is required for the full extension of K1-T6SS sheaths. (*A*) In vivo imaging of *P. putida rpoN* TagB1-sfGFP. TagB1 forms transient foci (white arrowhead). Disappearance of foci is not due to photobleaching, as new TagB1-sfGFP foci appear in the same or neighboring cells (red arrowheads). (*B*) Quantification of fluorescence intensity demonstrates that TagB1 foci accumulate over time before abruptly disappearing. Five representative foci are presented. The dotted line indicates the average background signal in the cytosol of bacteria in which no foci were detected during the course of imaging. Examples of 15 additional foci are shown in *SI Appendix*, Fig. S5. For *A* and *B*, the panels are selected images from fluorescence microscopy time-lapse recordings of *P. putida rpoN* expressing TagB1-sfGFP from the native *tagB1* locus (Movie S2); images were recorded every 2 s. (*C*) Formation of *P. putida* TagB1-sfGFP foci is dependent on TssA1. Shown are representative fluorescence microscopy images of *P. putida rpoN* and *P. putida rpoN tssA1* expressing TagB1-sfGFP from the native *tagB1* locus. (*D*) Quantification of images in *C*. (*Left*) Approximately 10% of cells form TagB1-sfGFP foci in *P. putida rpoN*, while no foci are present in the isogenic *tssA1* mutant. (*Right*) The number of cells containing TssA1-sfGFP foci remains unchanged on deletion of *tagB1*. *n* indicates the number of cells included in the analysis. (*E*) Deletion of *tagB1* does not affect the number of T6SS sheaths formed. As for *P. putida rpoN*, ∼10% of cells form sheaths in *P. putida rpoN tagB1*. Data shown for *P. putida rpoN* were also presented in Fig. 1*F*. *n* indicates the number of cells included in the analysis. (*F*) Absence of TagB1 prevents full extension of the K1-T6SS sheath. Shown are representative fluorescence microscopy images of *P. putida rpoN* and *P. putida rpoN tagB1* expressing TssB1-sfGFP from the native *tssB1* locus (see also *SI Appendix*, Fig. S6). (*G*) Quantification of *F*. The average sheath length in a *tagB1* mutant is ∼50% smaller than that in *P. putida rpoN*. Statistical analysis was performed using the unpaired *t* test with Welch’s correction. *P. putida rpoN* TssB1-sfGFP, *n* = 98; *P. putida rpoN tagB1* TssB1-sfGFP, *n* = 95; 174.8 degrees of freedom; *t* value = 16.71; *P* = 5.065 × 10^−38^ (significant; statistical significance is defined as *P* < 0.05 and indicated by ****P < 0.0001). (*H*) The cell width of a *tagB1* mutant is identical to that of the parental *P. putida rpoN* strain. Statistical analysis was performed using the unpaired *t* test with Welch’s correction; *P. putida rpoN* TssB1-sfGFP, *n* = 706; *P. putida rpoN tagB1* TssB1-sfGFP, *n* = 693; 1,389 degrees of freedom; *t* value = 0.2638; *P* = 0.7920. Data and images presented in *E–H* were generated from fluorescence microscopy time-lapse recordings of *P. putida rpoN* and its isogenic *tagB1* mutant expressing TssB1-sfGFP; images were recorded every 2 s. (Scales bars for *A–F*: 1 μm.)

Having confirmed that *P. putida* K1-T6SS sheaths display characteristic behaviors (Fig. 1 *D*–*G*), we proceeded to investigate the behavior of TagB1 in vivo. We generated a *P. putida rpoN* strain expressing TagB1 fused to sfGFP from the native *tagB1* locus and found that TagB1-sfGFP forms transient foci (Fig. 2*A*) that accumulate over time before abruptly disappearing (Fig. 2*B*, *SI Appendix*, Fig. S5, and Movie S2). Similar to sheaths (Fig. 1*F*), ∼10% of *P. putida rpoN* cells contain TagB1-sfGFP foci at any given time (Fig. 2*D*). The same transient foci were also detected in wild-type *P. putida* expressing TagB1-sfGFP (*SI Appendix*, Fig. S4*B*), but as expected, a markedly lower percentage of cells exhibited foci. Importantly, no TagB1-sfGFP foci were observed in the absence of TssA1 (Fig. 2 *C* and *D*, *Left*), while the number of cells exhibiting TssA1-sfGFP foci remained unchanged in a *tagB1* mutant (Fig. 2*D*, *Right*). This indicates that the interaction of TagB1 with TssA1 drives the formation of TagB1 foci, whereas the absence of TagB1 does not affect the behavior of TssA1.

TssA is known to promote T6SS assembly by initiating and orchestrating sheath polymerization (27). As *P. putida* TagB1 interacts with TssA1 (Fig. 1*C* and Dataset S2) and TagB1-sfGFP foci are dependent on TssA1 for their formation (Fig. 2 *C* and *D*), we hypothesized that TagB1 could be important for K1-T6SS sheath formation. We imaged sheath dynamics in a *tagB1* mutant expressing TssB1-sfGFP and found that the number of sheaths formed at any given time in this strain was comparable to that in *P. putida rpoN* (Fig. 2*E*). However, in the absence of *tagB1*, the sheath length was significantly reduced (Fig. 2 *F* and *G* and *SI Appendix*, Fig. S6), with sheaths produced by the *tagB1* mutant reaching only ∼50% of the length of those produced by the parental strain (Fig. 2*G*). Since cell width has been shown to control T6SS sheath length (33), we determined the average cell width in the *tagB1* mutant and found it to be identical to that of the parental strain (Fig. 2*H*). The fact that deletion of *tagB1* affects the maximum length of the sheath points to a role for TagB1 in sheath stabilization, similar to that of TagA in *E. coli*. Indeed, absence of the TagA anchor also affects the maximum length of the sheath, in this case leading to overly large sheaths that tend to break (30). Therefore, our results suggest that the K1-T6SS, which contains a short TssA, is stabilized through a small (∼30 kDa) structural component rather than a large TssA-like anchor protein, as is the case for long TssA proteins.

**Fig. 3.**
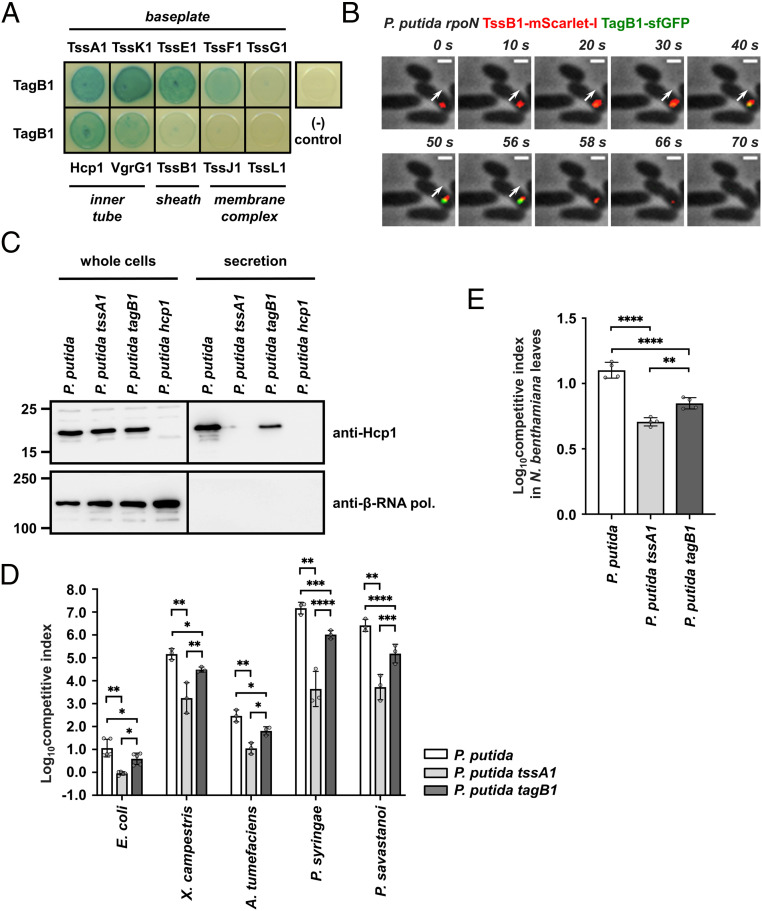
*P. putida* TagB1 stabilizes the K1-T6SS sheath from the baseplate, enabling optimized firing. (*A*) *P. putida* TagB1 interacts with all but one of the baseplate proteins but does not interact with sheath and membrane complex components, as assessed by bacterial two-hybrid assay. Three independent experiments were performed, with identical results. (*B*) *P. putida* TagB1 localizes to the baseplate. The white arrow indicates the direction of sheath extension. The panels are selected images from a fluorescence microscopy time-lapse recording of *P. putida rpoN* expressing TagB1-sfGFP from the native *tagB1* locus. The gene encoding TssB1-mScarlet-I was integrated into the chromosome while the native *tssB1* copy was still present. Images were recorded every 2 s (Scale bar: 1 μm.). See also *SI Appendix*, Fig. S7*B*. (*C*) The *tagB1* mutant of *P. putida* shows markedly reduced secretion of Hcp1. Hcp1 expression and presence in the culture supernatant were assessed for wild-type *P. putida* and the isogenic *tssA1* and *tagB1* mutants; the *P. putida hcp1* mutant was used as a negative control for Hcp1 detection. Positions of molecular weight markers are shown on the left. The β-subunit of the *E. coli* RNA polymerase (β-RNA pol.) was used as a loading and bacterial lysis control, and black lines indicate where the membrane was cut. A representative blot from three independent experiments is presented. (*D*) Competition assays between *P. putida* strains, *E. coli*, and a set of plant pathogens show decreased competitive index for the *tagB1* mutant compared to the wild-type *P. putida* strain. Data are mean ± SD. *P < 0.05; **P < 0.01; ***P < 0.001; ****P < 0.0001. Statistical analysis was performed using unpaired *t* tests corrected for multiple testing using the Holm–Sidak method. For *E. coli* comparisons, *n* = 5; *P. putida* vs. *P. putida tssA1*, adjusted *P* = 0.001167 (8 df); *P. putida* vs. *P. putida tagB1*, adjusted *P* = 0.019402 (24 df); *P. putida tssA1* vs. *P. putida tagB1*, adjusted *P* = 0.030542 (24 dt). For *A. tumefaciens* comparisons, *n* = 3; *P. putida* vs. *P. putida tssA1*, adjusted *P* = 0.006286 (4 df); *P. putida* vs. *P. putida tagB1*, adjusted *P* = 0.019402 (24 df); *P. putida tssA1* vs. *P. putida tagB1*, adjusted *P* = 0.030542 (24 df) (all significant). For *P. syringae* comparisons, *n* = 3; *P. putida* vs. *P. putida tssA1*, adjusted *P* = 0.006286 (4 df); *P. putida* vs. *P. putida tagB1*, adjusted *P* = 0.000135 (24 df); *P. putida tssA1* vs. *P. putida tagB1*, adjusted *P* < 0.000001 (24 df) (all significant). For *P. savastanoi* comparisons, *n* = 3; *P. putida* vs. *P. putida tssA1*, adjusted *P* = 0.006286 (4 df); *P. putida* vs. *P. putida tagB1*, adjusted *P* = 0.000069 (24 df); *P. putida tssA1* vs. *P. putida tagB1*, adjusted *P* = 0.000409 (24 df) (all significant). (*E*) In planta competition assay between *P. putida* strains and the phytopathogen *X. campestris* show decreased competitive index for the *tagB1* mutant compared to the wild-type *P. putida* strain. Data are mean ± SD. *P < 0.05; **P < 0.01; ***P < 0.001. Statistical analysis was performed using one-way ANOVA with Tukey’s multiple comparison test; *n* = 4; 11 df; *F* = 73.55; *P* < 0.0001 (significant). *P. putida* vs. *P. putida tssA1*, adjusted *P* < 0.0001; *P. putida* vs. *P. putida tagB1*, adjusted *P* < 0.0001; *P. putida tssA1* vs. *P. putida tagB1*, adjusted *P* = 0.0051 (all significant).

TagB1 interacts with TssA1 (Fig. 1*C* and Dataset S2), and thus it is plausible that it could stabilize the T6SS apparatus from either end of the sheath, since TssA1 localizes initially at the baseplate of the system before being displaced to the other side of the cell via sheath extension. Bacterial two-hybrid analysis showed that TagB1 can interact with all but one of the baseplate proteins (TssK1, TssE1, TssF1, Hcp1, VgrG1, and TssA1) but does not interact with representative components of the sheath (TssB1) or the membrane complex (TssJ1 and TssL1) (Fig. 3*A*). It is likely that TagB1 interacts with TssA1 with higher affinity compared to other baseplate components, as indicated by the fact that only TssA1 copurified with TagB1 (Dataset S2) and supported by the observation that TssA1 is essential for TagB1-sfGFP focus formation (Fig. 2 *C* and *D*) and thus its localization to the T6SS apparatus. In agreement with the bacterial two-hybrid data, when we simultaneously visualized TagB1 and TssB1 in vivo, TagB1-sfGFP foci were associated with the extremity of the extended sheath toward which contraction occurred (*SI Appendix*, Fig. S7*A*). This suggests that TagB1 localizes at the baseplate. Further analysis of the full sheath extension and contraction cycle confirmed this. Once sheath polymerization started, TagB1-sfGFP foci appeared and accumulated at the site of sheath initiation (Fig. 3*B* and *SI Appendix*, Fig. S7*B*; sheath extension, 0 to 56 s; TagB1-sfGFP focus appearance, 10 s), and the disappearance of TagB1-sfGFP foci coincided with sheath contraction (Fig. 3*B* and *SI Appendix*, Fig. S7*B*; sheath contraction, 58 to 70 s). Together these results (Fig. 3 *A* and *B* and *SI Appendix*, Fig. S7) show that TagB1, unlike TagA, localizes at the baseplate and not at the distal end of the sheath. This conclusion is further supported by the fact that after a contraction event, TagB1-sfGFP foci occasionally reappeared in the same position in the cell (*SI Appendix*, Fig. S8). This is much more likely to occur if TagB1 associates with the baseplate rather than with the distal end of the sheath and is consistent with the reuse of T6SS membrane complex structures observed by Zoued et al. (27).

Taken together, our data reveal a previously unidentified mode of stabilization for this TssA_S_-containing T6SS. In brief, we show that TagB1 is a structural T6SS protein that stabilizes sheath polymerization from the baseplate (Fig. 3 *A* and *B*) and enables the sheath to reach its full size (Fig. 2 *F*–*H*). For this to happen, TagB1 is recruited to the baseplate by TssA1 (Dataset S2 and Figs. 1*C* and 2 *C* and *D*), where it continues to accumulate (Figs. 2*B* and 3*A* and *B*, *SI Appendix*, Fig. S5, and Movie S2) through self-interaction (Fig. 1*C*), supporting the polymerizing sheath after TssA1 has left the site of sheath initiation for the end of the extending structure (Fig. 1*G*).

### TagB1-Mediated Sheath Stabilization Allows Optimal K1-T6SS Firing.

Having determined that TagB1 plays a stabilizing role in assembly of the T6SS sheath, we investigated the effect of its absence on K1-T6SS function and during interbacterial competition. We found that compared to a wild-type strain, a *tagB1* mutant exhibited substantially decreased Hcp1 secretion (Fig. 3*C*), which could be restored to wild-type levels in a complemented strain (*SI Appendix*, Fig. S9). Consistent with this, the *tagB1* mutant has a significantly reduced competitive index when cocultured with preys such as *E. coli* or a range of plant pathogens that *P. putida* would encounter and compete with in its natural environments (32) (Fig. 3*D*). We observed the same effect during in planta competition of *P. putida* strains with the plant pathogen *Xanthomonas campestris* (performed in *Nicotiana benthamiana* leaves) (Fig. 3*E*). For all assays, the *tagB1* phenotype was not as drastic as that of a *tssA1* mutant, resulting in an entirely inactive T6SS (32) (Fig. 3 *C–E*). This is in agreement with the ability of the *tagB1* mutant strain to form sheaths, which, although shorter (Fig. 2 *F* and *G*), are likely to still deliver some effector cargo. Thus, while TagB1 is not essential for T6SS activity, it is still a key component of the system, mediating optimal firing of the K1-T6SS and effective killing of prey bacteria through its stabilizing role.

### TagB and TagJ Components Produce a Distinct Stabilization Mode for T6SSs with Short TssA Proteins.

We have demonstrated that the *P. putida* K1-T6SS is stabilized and achieves optimal function through the recruitment of the small protein TagB1 to the baseplate by TssA1 (a TssA_S_ protein). To determine if this mode of sheath stabilization applies to other T6SSs encoding short TssA components, we selected the H1-T6SS from *P. aeruginosa*, which belongs to phylogenetic group 3 and contains the putative accessory protein TagJ1 (Fig. 1*B* and Dataset S1). We performed dot blot assays with pure TagJ1-StrepII to assess both self-interaction and interaction with its cognate TssA_S_ protein. Like *P. putida* TagB1, TagJ1 self-interacts and binds strongly to TssA1 (*SI Appendix*, Fig. S10*A*), and these associations are specific to TagJ1 and TssA1, as no interactions were detected when using a binding control protein or lysates from cells harboring the empty vector (*SI Appendix*, Fig. S10*A*). In addition, TagJ1-sfGFP forms transient foci in vivo (*SI Appendix*, Fig. S10*B*) which, in a similar manner to *P. putida* TagB1-sfGFP foci, remain static before abruptly disappearing. Therefore, while TagJ1 and TagB1 are not homologous, our data indicate that they likely perform similar functions during T6SS assembly. As such, the sheath-stabilizing role that we describe for *P. putida* TagB1 likely extends to other T6SSs encoding short TssA proteins associated with TagB, TagJ, or other similar-sized partners (Fig. 1*B*). This mechanism of T6SS stabilization, via baseplate-associated 30-kDa components, is completely distinct from the previously reported TagA-mediated anchoring (29, 30) that secures the sheath through clamping of its distal end.

### Distinct Stabilization Modes Generate Functionally Diverse T6SSs.

**Fig. 4.**
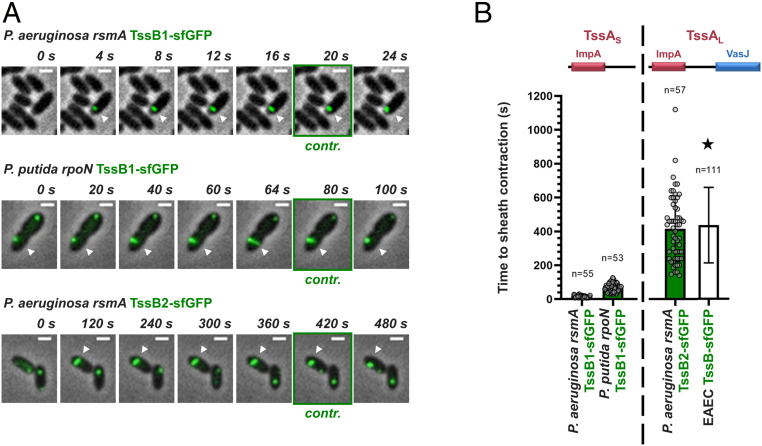
Distinct stabilization modes correlate with different T6SS behaviors. (*A*) T6SSs with short TssA proteins and TagB or TagJ accessory components contract immediately after sheath extension; examples presented here are the *P. aeruginosa* H1-T6SS (TagJ-mediated sheath stabilization) and the *P. putida* K1-T6SS (TagB-mediated sheath stabilization) (*Top* and *Middle*, respectively). T6SSs with long TssA proteins reside for prolonged periods in the cell after sheath extension; the example shown here is the *P. aeruginosa* H2-T6SS (*Bottom*). The panels are selected images from fluorescence microscopy time-lapse recordings of *P. putida rpoN* expressing TssB1-sfGFP from the native *tssB1* locus and *P. aeruginosa rsmA* expressing TssB1-sfGFP and TssB2-sfGFP from the native *tssB1* and *tssB2* loci, respectively. White arrowheads indicate the T6SS sheaths of interest, and panels showing sheath contraction (“contr.”) are marked with a green outline. Images were recorded every 2 s for the H1- and K1-T6SS and every 20 s for the H2-T6SS. (Scale bars: 1 μm.) (*B*) Quantification of *A* and integration of the data collected as part of this study with measurements from the literature. Time to sheath contraction was measured for the T6SSs shown in *A*. Values for the T6SS of enteroaggregative *E. coli* (EAEC) (30), marked by a star, refer to residence times only. *n* indicates the number of cells included in the analysis.

*E. coli* TagA, which associates with a TssA_L_ protein, has been shown to maintain the assembled sheath in the extended conformation for long periods (30, 34). Moreover, the TssA_L_-containing H2-T6SS from *P. aeruginosa* has also been reported to be stable in its extended conformation (29). This contrasts with T6SSs that contain short TssA proteins, like the *P. aeruginosa* H1-T6SS, which is known for its rapid firing (29, 35), as well as the *P. putida* K1-T6SS, which we found to fire immediately after sheath extension (Movie S1). Knowing that the aforementioned T6SS apparatuses have different stabilization mechanisms (TssA_L_-TagA or TssA_S_-TagB/J), we assessed the correlation between the mode of T6SS stabilization, determined primarily by the type of TssA (TssA_L_ or TssA_S_) and the nature of its associated stabilizing protein, and the time from the initiation of sheath polymerization until its contraction. To do this, we systematically measured the time to contraction of T6SSs from different phylogenetic groups (*P. aeruginosa* H1-T6SS, group 3; *P. putida* K1-T6SS; group 4B; *P. aeruginosa* H2-T6SS, group 1) (Fig. 4*A*) and integrated our data with values from the literature (*E. coli* T6SS, group 2) (30) (Fig. 4*B*). We show that T6SSs with TssA_S_ proteins and a TagB or TagJ partner contract immediately after sheath extension, taking between 20 and 70 s to assemble and fire, whereas systems with TssA_L_ proteins tend to reside in the cell (Fig. 4*B*). Our own time to contraction measurements on the *P. aeruginosa* H2-T6SS are largely comparable with the measurements of sheath residence time for the *E. coli* T6SS (30) and show that these systems can remain extended and poised to fire for longer than 10 min (Fig. 4*B*). Moreover, while comprehensive measurements for the time to contraction of the *V. cholerae* T6SS (TssA_L_/TagA_V_ anchor; group 1) have not been performed, residence times of up to 220 s have been reported (29). Notably, although the stability of TssA_L_-containing T6SSs is usually supported by TagA anchors (29, 30, 34), it has been postulated that TssA_L_ components could act as anchors themselves—for example, in the case of *P. aeruginosa* H2-T6SS (29), for which no TagA protein has been identified to date (Dataset S1). This suggestion is supported by our results showing that *P. aeruginosa* H2-T6SS sheaths are stably anchored (Fig. 4*A*, bottom row). Overall, our results show that the type of TssA (TssA_L_ or TssA_S_) determines the firing dynamics of a T6SS apparatus through the recruitment of its respective sheath stabilizing protein partner (TagA or TagB/J). As such, the specific pairing of these two structural components likely underpins the different aggression strategies observed across T6SS-carrying bacteria.

## Discussion

**Fig. 5.**
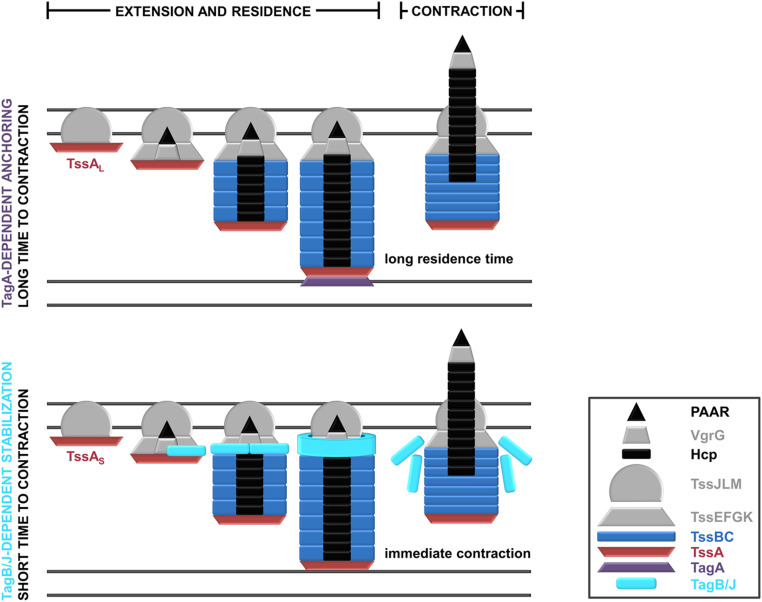
Schematic representation of the characterized modes of T6SS stabilization. (*Top*) T6SSs containing TssA_L_ proteins are usually anchored by TagA structural components. The TagA anchor is recruited by TssA_L_ as the latter approaches the opposing cell membrane and clamps the end of the sheath, stabilizing it for prolonged periods. (*Bottom*) T6SSs containing TssA_S_ proteins are stabilized by protein partners like TagB or TagJ, which are recruited to the baseplate by TssA. As the sheath extends with TssA at its distal end, TagB/TagJ continues to accumulate on the baseplate, forming a collar-like structure that stabilizes the extended conformation of the T6SS apparatus and allows the sheath to reach its full size. In this stabilization mode, T6SS firing occurs immediately after the sheath reaches the opposing cell membrane. (*Inset*) A key to the colors and shapes used to depict T6SS protein components.

In this work, we report and characterize a yet undescribed T6SS stabilization mode used by apparatuses containing short TssA proteins. This mode of sheath stabilization relies on the TssA-dependent recruitment of a class of protein partners (TagB or TagJ) to the baseplate structure. These T6SS components do not necessarily share any sequence similarity but have nearly identical molecular weights (Fig. 1*B*). Our findings provide much-needed insight into the assembly of TssA_S_-containing T6SSs and allow us to propose an overall framework for T6SS sheath stabilization (Fig. 5). TssA_L_-containing T6SSs usually rely on membrane-associated TagA components for anchoring. TagA proteins interact with TssA_L_, stably clamping the end of the sheath at the opposite side of the cell and maintaining the sheath in the extended conformation (Fig. 5, *Top*). Absence of TagA allows the end of the sheath to slide and the structure to keep elongating, which delays contraction even further and often results in broken or floating sheaths of unnatural length (29, 30, 34). In contrast, T6SSs with TssA_S_ proteins have no terminal sheath anchor and are instead stabilized by the recruitment and accumulation of TagB or TagJ structural components at the baseplate (Fig. 3 *A* and *B*). One can envisage that these support proteins form a collar-like structure that constricts the baseplate (Fig. 3 *A* and *B*), preventing the conformational change (dome- to star-shaped baseplate) that leads to contraction (36) until the sheath polymerizes to its full size (Fig. 2 *F*–*H*). The sheath contracts as soon as it reaches the opposing cell membrane (Figs. 1*D* and 3*B*), dispersing the TagB or TagJ multimer (Fig. 5, *Bottom*). Both modes of sheath stabilization are important for T6SS efficacy in interbacterial killing, as loss of either type of stabilizing protein leads to increased prey survival (30) (Fig. 3 *D* and *E*). For TagA-mediated anchoring, decreased prey killing is due to unchecked polymerization and breaking of the sheaths (30). By contrast, for TagB1-mediated stabilization, the production of overly short sheaths results in a reduction in prey killing (Figs. 2 *F* and *G* and 3 *D* and *E*). This may be due to the delivery of fewer Hcp-associated effectors (lower *P. putida* Hcp1 secretion; Fig. 3*C*), reduced mechanical energy of the sheath, or, most likely, a combination of both. We note that three orphan *hcp* clusters, each associated with potential effector-immunity pairs, have been identified bioinformatically in *P. putida* (32). These additional Hcp proteins (Hcp4, Hcp5, and Hcp6) are phylogenetically related to Hcp1 (*SI Appendix*, Fig. S11) and likely secreted by the K1-T6SS, further supporting this hypothesis.

We have shown that the pairing of different forms of TssA (TssA_L_ or TssA_S_) with diverse accessory proteins (TagA or TagB/J) generates distinct T6SS stabilization modes which correlate with longer or shorter times to sheath contraction (Fig. 4). Varied timescales of sheath assembly and firing naturally result in very different numbers of assembled T6SS apparatuses per cell. In cases where the sheath is stabilized via a TssA_S_-TagB/J mechanism, the vast majority of cells contain a single sheath at any given time, whereas in systems anchored by TssA_L_-TagA, cells usually contain more than one sheath (29, 33) and can harbor up to six fully assembled structures (*SI Appendix*, Fig. S12). Such diverse numbers of T6SSs per cell can generate various aggression strategies. For T6SSs with short residence times, cells can engage in constant firing (*P. putida* K1-T6SS; ref. 32) or quickly retaliate against incoming attacks (*P. aeruginosa* H1-T6SS; ref. 35), while for T6SSs with long residence times, attackers can deploy a larger-than-normal effector payload on a specific cue through the firing of multiple apparatuses per cell.

The description of T6SS sheath stabilization by TagB or TagJ proteins raises the question of how sheath stabilization occurs in T6SSs containing a TssA_S_ protein but no identified anchoring partner; for example, T6SSs belonging to phylogenetic group 4A (Fig. 1*B*), like the *P. aeruginosa* H3-T6SS. To begin to probe this, we generated a *P. aeruginosa rsmA* strain expressing TssB3-sfGFP from the native *tssB3* locus and imaged the H3-T6SS sheaths. We found sheath assembly in this system to be markedly different from many of the T6SSs visualized previously. TssB3-sfGFP foci and assembled sheaths were always located at the cell pole (*SI Appendix*, Fig. S13*A*), which was not observed for the *P. aeruginosa* H1- and H2-T6SSs (*SI Appendix*, Fig. S13*B*). H3-T6SS sheaths contract (*SI Appendix*, Fig. S13*C*), but instead of polymerizing across the short axis of the cell like other sheaths imaged in this study, they extend from the cell pole. In particular, they either form across the long axis of the cell, leading to apparatuses with a “floating” end (*SI Appendix*, Fig. S13*A*, red arrowhead), or extend diagonally, eventually leaning against the opposing cell wall (*SI Appendix*, Fig. S13*A*, yellow arrowhead). This distinctive behavior seems to be in agreement with the lack of an obvious sheath anchor (Fig. 1*B*) and alludes to a third mode of sheath stabilization that correlates with polar localization of the T6SS apparatus. The lack of a stabilization mechanism could lead to suboptimal firing for the *P. aeruginosa* H3-T6SS. Nonetheless, as no toxins have been associated with this system to date (the H3-T6SS has only been shown to secrete an effector involved in iron acquisition; ref. 6), it may be that the function of this system is to allow the secretion of common goods effectors to the extracellular milieu. In this case, optimal firing probably is not required. The polar localization of the system possibly allows it to be poised and ready to fire when iron becomes limited without obstructing the assembly of the other two T6SSs of this bacterium (37), which usually are not localized at the pole (*SI Appendix*, Fig. S12*A*).

Overall, the distinct firing modalities that emerge from the different T6SS stabilization mechanisms described here could benefit bacteria in varied competition scenarios and ecological niches. This suggests that the functional diversity of T6SS apparatuses not only stems from the broad range of effectors that they can deliver (38, 39), but also can be generated by differences in the structural components that underpin the modes and dynamics of T6SS assembly and firing.

## Materials and Methods

Detailed descriptions of the methods used for genetics, mass spectrometry analysis, microbiological assays (interbacterial competition and T6SS assays), protein-protein interaction experiments (dot blot and bacterial two-hybrid assays), bioinformatics analyses, and microscopy experiments, along with associated image analyses, can be found in the *SI Appendix*. Unless otherwise indicated, lysogeny broth (LB) (10 g/L NaCl) and agar (1.5% wt/vol) were used for routine growth of all organisms with shaking at 200 RPM, as appropriate. *E. coli* and *P. aeruginosa* were grown at 37 °C, *P. putida* at 30 °C, and plant pathogens at 28 °C. For competition assays with plant pathogens, LB (5 g/L NaCl) was used, whereas bacterial cultures for microscopy experiments and secretion assays were grown using tryptone soya broth (Oxoid). Imaging was performed with a Zeiss Axio Observer Z1 inverted widefield microscope equipped with a Plan-Apochromat 63×/1.4 NA Oil Ph3 M27 objective (Zeiss), a SpectraX LED light engine (Lumencore), an ORCA-Flash 4.0 digital CMOS camera (Hamamatsu), and an environmental control system. Generation of images was performed using the ZEN Blue software (Zeiss) and FIJI (40), and image processing and analysis were performed using FIJI in conjunction with the MicrobeJ (41) plugin. Microscopy was performed at the Facility for Imaging by Light Microscopy at Imperial College London.

## Supplementary Material

Supplementary File

Supplementary File

Supplementary File

Supplementary File

Supplementary File

## Data Availability

All study data are included in the main text and/or supporting information.
