# Supplementary material for: A novel stabilization mechanism for the type VI secretion system sheath
Source: Proc Natl Acad Sci U S A. 2021 Feb 8;118(7):e2008500118. doi: 10.1073/pnas.2008500118 (PMC7896307; doi:10.1073/pnas.2008500118)
Supplement: Supplementary File [file pnas.2008500118.sapp.pdf]

SUPPLEMENTARY INFORMATION (SI) APPENDIX FOR

A novel stabilization mechanism for the type VI secretion system sheath

Patricia Bernal[a,c,d,2], R. Christopher D. Furniss[a,2], Selina Fecht[a], Rhoda C.Y. Leung[a], Livia Spiga[a], Despoina A.I. Mavridou[a,b,1], Alain Filloux[a,1]

[a]MRC Centre for Molecular Bacteriology and Infection, Department of Life Sciences, Imperial College London, London, SW7 2AZ, UK
[b]Department of Molecular Biosciences, University of Texas at Austin, Austin, 78712, Texas, USA
[c]Department of Biology, Faculty of Sciences, Universidad Autónoma de Madrid, Madrid, 28049, Spain
[d]Departamento de Microbiología, Facultad de Biología, Universidad de Sevilla, Seville, 41012, Spain

[1]To whom correspondence should be addressed. A. Filloux, MRC Centre for Molecular Bacteriology and Infection, Department of Life Sciences, Imperial College London, London, SW7 2AZ, UK, Tel: +44(0)2075949651, E-mail: a.filloux@imperial.ac.uk; D.A.I. Mavridou, Department of Molecular Biosciences, University of Texas at Austin, Austin, 78712, Texas, USA, Tel: +15124756864, E-mail: despoina.mavridou@austin.utexas.edu

[2]These authors have contributed equally to this work.

This PDF file includes:

Supplementary Materials and Methods
Figures S1 to S14
Tables S1 to S3
Legends for Videos S1 and S2
Legends for Dataset S1 and S2
Supplementary references

SUPPLEMENTARY INFORMATION MATERIALS AND METHODS

Reagents and bacterial growth conditions. Unless otherwise stated, chemicals, antibiotics and reagents were acquired from Sigma Aldrich and growth media were purchased from Merck or Oxoid. Lysogeny broth (LB) (10 g/L NaCl) and agar (1.5% w/v) were used for routine growth of all organisms with shaking at 200 RPM, as appropriate; *Escherichia coli* and *Pseudomonas aeruginosa* were grown at 37 ºC, *Pseudomonas putida* at 30 ºC and plant pathogens (*Xanthomonas campestris, Agrobacterium tumefaciens, Pseudomonas syringae* and *Pseudomonas savastanoi*) at 28 ºC. For competition assays with plant pathogens LB (5 g/L NaCl) was used, whereas bacterial cultures for microscopy experiments and secretion assays were grown using tryptone soya broth (TSB) (Oxoid). Growth media were supplemented with the following, as required: 1 mM Isopropyl β-D-1-thiogalactopyranoside (IPTG), 30 µg/mL tetracycline, 100 µg/mL ampicillin, 50 µg/mL kanamycin, 20 µg/mL gentamicin, 30 µg/mL chloramphenicol, 25 µg/mL piperacillin, 20 µg/mL rifampicin, 100-200 µg/mL streptomycin for *P. putida*, and 2000 µg/mL streptomycin for *P. aeruginosa*.

Construction of plasmids and bacterial strains. Bacterial strains, plasmids and oligonucleotides used in this study are listed in Tables S1, S2 and S3, respectively. DNA manipulations were conducted using standard methods. KOD Hot Start DNA polymerase (Merck) was used for all PCR reactions according to the manufacturer's instructions, oligonucleotides were synthesized by Sigma Aldrich and restriction enzymes were purchased from Roche and New England Biolabs. All constructs were DNA sequenced and confirmed to be correct before use. Recombinant plasmids were transferred to *E. coli* strains by transformation and to *Pseudomonas* strains by electroporation (1) or conjugation (2), as appropriate.

Genes encoding T6SS structural components (*P. putida* Hcp1, TssA1, TagB1, TssB1, TssE1, TssF1, TssG1, TssJ1, TssK1, TssL1 and VgrG1 as well as *P. aeruginosa* TssA1 and TagJ1) were amplified from genomic DNA extracted from *P. putida* KT2440R and *P. aeruginosa* PAO1. *P. putida hcp1* was cloned into the IPTG-inducible plasmid pET28a using primers P1 and P2; when expressed, *P. putida* Hcp1 has an N-terminal His_6 tag. *P. putida tssA1* and *tagB1* and *P. aeruginosa tssA1* and *tagJ1* were cloned into the IPTG-inducible plasmid pETDuet-1, in MCS-1 (*tssA1*) and MCS-2 (*tagB1* and *tagJ1*) using primers P3-P18. When expressed, *P. putida* and *P. aeruginosa* TssA1 proteins have an N-terminal His_6 tag (genes cloned with primer pairs P5/P6 and P9/P10, respectively) or an N-terminal V5 tag (GKPIPNPLLGLDST) (primer pairs P11/P12 and P15/P16, respectively were used for exchange of the affinity tag). When expressed, TagB1 and TagJ1 have a C-terminal StrepII tag (genes cloned with primer pairs P3/P4 and P7/P8, respectively) or V5 tag (primer pairs P13/P14 and P17/P18, respectively were used for exchange of the affinity tag). *P. putida tssE1*, *tssF1*, t*ssG1*, *tssK1*, *hcp1*, *vgrG1*, *tssA1*, *tssB1*, *tssJ1* and *tssL1* were cloned into the bacterial-two-hybrid vector pKNT25 using primers P68-P87 in order to generate chimeric genes encoding N-terminal fusions of the respective proteins with the T25 fragment of *Bordetella pertussis* adenylate cyclase. *P. putida tagB1* was cloned into vectors pUT18C (primer pair P64/65) and pUT18 (primer pair P66/67) to produce chimeric genes encoding C- and N-terminal fusions of TagB1 with the T18 fragment of *B. pertussis* adenylate cyclase.

P. putida gene mutants (*tagB1* and *hcp1*) were constructed by allelic exchange, as previously described (3). Briefly, 500-bp DNA fragments upstream and downstream the gene to be deleted were amplified using *P. putida* KT2440 genomic DNA. A fragment containing both regions was obtained by overlapping PCR, cloned into pCR-BluntII-TOPO (Invitrogen),

DNA sequenced and subcloned into the *Xba*I/*Bam*HI sites of pKNG101 (primers P19-P22 (for *tagB1*) and P98-P101 (for *hcp1*)). The suicide vector pKNG101 (4) does not replicate in *Pseudomonas*; it was maintained in *E. coli* CC118λpir and mobilized into *Pseudomonas* by triparental conjugation. The same method was employed to re-introduce *tagB1* in its native locus in *P. putida tagB1*, in order to complement the mutant strain. In particular, a DNA fragment containing *tagB1*, flanked by 500 bp upstream and downstream, was amplified using *P. putida* KT2440 genomic DNA and primers P19 and P22. This was then cloned into pCR-BluntII-TOPO (Invitrogen), DNA sequenced, subcloned into the *Xba*I/*Bam*HI sites of pKNG101, and mobilized into *P. putida tagB1* by triparental conjugation

A similar approach was used to replace wild-type *P. putida tssA1*, *tssB1* and *tagB1* as well as *P. aeruginosa tssB1*, *tssB2*, *tssB3* and *tagJ1* with genes encoding the protein of interest C- or N-terminally fused to a sfGFP or mScarlet-I fluorophore (all fusions carry the fluorophore at the C-terminus of the protein with the exception of the *P. putida* TssA1 fusion where the sfGFP is at the N-terminus of the protein); primers P23-P43, P46-P63 and P88-P93 were used for engineering these substitutions. The same strategy was used to replace the wild-type *P. putida tagB1* gene with a version encoding TagB1 C-terminally fused to two consecutive StrepII tags (primers P94-P97). Finally, the *tssB1*-mScarlet-I gene (amplified with primer pair P44/P45), encoding a C-terminal fusion of m-Scarlet-I to *P. putida* TssB1, was introduced on the chromosome using the miniCTX vector (5). All insertions and gene replacements were confirmed by PCR and DNA sequencing.

Protein identification by mass spectrometry. Bacterial cultures of *P. putida rpoN* and of a *P. putida rpoN* strain where *tagB1* was replaced with a version of the gene encoding TagB1 C-terminally fused to two consecutive StrepII tags, were grown in TSB supplemented with the appropriate antibiotics for at least 8 hours at 30 ºC with shaking at 200 RPM. Bacterial suspensions were then sub-cultured at an OD_{600} of 0.1 into 50 ml TSB and incubated for an additional 8 hours under the same growth conditions. Cells were harvested and cell pellets were resuspended in 50 mM Tris-HCl (pH 7.5), 150 mM NaCl and lysed by sonication after the addition of protease inhibitors (Roche). Cell debris was eliminated by centrifugation (48,000 *x* g, 30 mins, 4 °C) and both suspensions were subjected to protein purification using Strep-Tactin Sepharose (Iba Lifesciences), according to the manufacturer's specifications. The resulting protein mixtures were methanol precipitated and freeze dried; three samples were prepared for each purification condition.

Freeze-dried protein mixtures were resuspended to a final concentration of 1 μg/mL in 25 μL of 20 mM ammonium bicarbonate before overnight digestion with trypsin at 30 °C (ratio 20:1 protein mixture:trypsin). Direct analysis of peptide mixtures was carried out under trap and elute conditions using an Acclaim Pepmap 100 Nano-Trap and column (Thermo Fischer Scientific) on an Eksigent nanoLC Ultra 2D HPLC coupled to a Triple-TOF 5600+ mass spectrometer via a Nanospray III source (all AB Sciex). The peptides were loaded in 98% v/v water, 2% v/v acetonitrile, 0.05% v/v trifluoroacetic acid and washed on the trap for 20 minutes, before being injected onto the column and eluted over 100 minutes with a rising gradient of acetonitrile composed of solutions A and B (solution A = 98% v/v water, 2% v/v acetonitrile, 0.1% v/v formic acid; solution B = 98% v/v acetonitrile, 2% v/v water, 0.1% v/v formic acid) as follows: 0 minutes, 98%A:2%B; 60 minutes, 80%A:20%B; 75 minutes, 60%A:40%B; 80 minutes, 2%A:98%B, 87.5 minutes, 98%A:2%B, 100 minutes, 98%A:2%B. Mass spectra were acquired between 400-1250 *m/z* and the top 20 peptides with a charge between +2 and +5 were selected to proceed to MS/MS (95-1800 *m/z*). All mass

spectrometry was performed at the BBSRC Mass Spectrometry and Proteomics Facility at the University of St. Andrews, UK.

MaxQuant analysis. Data were processed using MaxQuant version 1.5.8.3 (6). Peptides were identified from MS/MS spectra searched against the Uniprot *P. putida* ATCC47054/DSM6125/NCIMB11950/KT2440 reference proteome (proteome ID: UP000000556) (accessed November 2019) using the Andromeda search engine (7). Carbamidomethylation, methionine oxidation and N-terminal acetylation were specified as variable modifications. *In silico* digest of the reference proteome was performed using the Trypsin/P setting with up to two missed cleavages allowed. The false discovery rate (FDR) was set at 0.01 for peptides, proteins and sites. The "re-quantify" function was enabled. The sequence decoy mode used was "revert". Protein quantification was performed using the MaxLFQ algorithm within MaxQuant (8). Unique and razor peptides were used for quantification. All other parameters were used as pre-set in MaxQuant.

Perseus analysis. Data were analyzed using Perseus version 1.5.8.5 (9). Proteins present in the "reverse", "only identified by site" and "potential contaminant" databases were removed, and proteins identified by one or more unique peptides retained for further analysis. LFQ intensities were logarithmized (log_2) and replicates grouped together before the data was filtered to retain only proteins identified in two or more replicates from at least one sample. Missing log_2 LFQ intensities were inferred using a downshifted normal distribution (1.8 downshift, 0.3 width) and proteins that were significantly enriched by TagB1-(StrepII)$_2$ identified using a two-sample *t*-test (permutation based false discovery rate (FDR) = 250, FDR = 0.05, S_0 = 0.2).

Dot blot assays. *P. putida* TagB1 and *P. aeruginosa* TagJ1 self-interaction, as well as interaction of these proteins with their cognate TssA1 proteins were assessed using dot blots as follows: *E. coli* BL21(DE3) cells expressing TagB1-StrepII, TagJ1-StrepII or a leaderless version of the *E. coli* cytochrome *c* maturation protein CcmG fused to a C-terminal StrepII tag (binding control protein) were grown in LB (10 g/L NaCl) supplemented with the appropriate antibiotics at 37 °C with shaking at 200 RPM to an OD_{600} of 0.8. Expression was subsequently induced using IPTG for 16 hours at 30 °C. Cells were harvested and cell pellets were resuspended in 50 mM Tris-HCl (pH 7.5), 150 mM NaCl and lysed by sonication after the addition of protease inhibitors (Roche). Cell debris were eliminated by centrifugation (48,000 *x* g, 30 mins, 4 °C) and proteins were purified using Strep-Tactin Sepharose (Iba Lifesciences) according to the manufacturer's specifications. 3.5 ng of pure TagB1-StrepII, TagJ1-StrepII and CcmG-StrepII were spotted onto Amersham Protran nitrocellulose membranes (0.45 µm pore size, GE Life Sciences) and spots were dried at room temperature. Membranes were blocked overnight at 4 °C in 3% w/v Bovine Serum Albumin (BSA)/TBS-T (0.1% v/v Tween 20). The following day, 150 OD_{600} units of *E. coli* BL21 (DE3) containing the empty vector (pETDuet-1) or over-expressing TagB1-V5, TagJ1-V5, *P. putida* V5-TssA1 or *P. aeruginosa* V5-TssA1 were resuspended in 10 mL of binding buffer (20 mM Tris-HCl (pH 8.0), 10% v/v glycerol, 100 mM NaCl, 3% w/v BSA) and lysed by sonication. Crude lysates, or 10 mL of binding buffer alone, were applied directly to the blocked nitrocellulose membranes before overnight incubation at 4 °C. Membranes were washed three times for 10 minutes with TBS-T and probed with mouse anti-V5 (Invitrogen) (dilution 1:5,000 in 3 w/v % BSA/TBS-T) or Strep-Tactin-HRP conjugate (Iba Lifesciences) (dilution 1:3,000 in 3 w/v % BSA/TBS-T) at room temperature for 4 hours. Goat anti-mouse IgG-HRP conjugate (Sigma Aldrich) (dilution 1:6,000 in 3% w/v BSA/TBS-T) was applied for 1 hour at room temperature, as appropriate. Membranes were washed three times for 10 minutes with TBS-T

prior to development with Immobilon Classico Western HRP substrate (Merck Millipore) using a Gel DOC XR+ Imager (Bio-Rad). The experiments generating the data presented in Fig. 1C and S10A were performed simultaneously and development of the membranes was carried out on the same day for all eight membranes shown. The presented membranes are uncut hence the same exposure time was applied for each of the spotted proteins on each membrane. For membranes incubated with empty vector lysates, the longest of the exposure times was applied during development to ensure that signal detection is maximized.

Interbacterial competition assays. *In vitro* competition assays were performed on LB (10 g/L NaCl) (*E. coli* prey) or LB (5 g/L NaCl) (plant pathogens as prey) agar (1.5% w/v) plates, as previously described (10). Briefly, overnight bacterial cultures were washed and adjusted to an OD_{600} of 10 in sterile PBS, and mixed at a 1:1 ratio (*P. putida*:prey). Mixtures were grown on LB agar plates at 30 °C for 5 hours (*E. coli* prey) or 24 hours (plant pathogens as prey) and then collected using an inoculating loop and resuspended in sterile PBS. The outcome of the competition was quantified by counting colony forming units (CFUs) using antibiotic selection of the input (time = 0 hours) and output (time = 5 hours or time = 24 hours). All prey strains harboured the plasmid pRL662, which confers resistance to gentamicin and was used for antibiotic selection, whereas *P. putida* KT2440R is naturally resistant to rifampicin. *In planta* competition assays were carried out by infiltration of bacteria into *Nicotiana benthamiana* leaves, as described before (11). Briefly, overnight cultures of *P. putida* and *X. campestris* were adjusted to OD_{600} of 0.1 in PBS and mixed at a 1:1 ratio. Approximately 100 µl of each suspension were infiltrated on the reverse of an approximately one-month-old leaf and the infiltration area was marked. After 24 hours of incubation in a plant chamber (23°C, 16 hours light exposure), a circular section of 6 mm diameter from the infiltration area of the leaf was isolated using a cork-borer set (Sigma-Aldrich), homogenized in PBS using a motorized tissue grinder (FisherBrand), serially diluted and platted on LB agar supplemented with the appropriate antibiotics. CFUs were determined using antibiotic selection as described above. For all competition assays, at least three biologically independent experiments were performed. Competitive index values were calculated using the following formula: competitive index = (output attacker/output prey)/(input attacker/input prey).

P. putida Hcp1 antibody production: An overnight TSB culture of *E. coli* BL21(DE3) cells harboring a pET28a plasmid encoding *P. putida* Hcp1 with an N-terminal His_6 tag was used to inoculate 1L of TSB supplemented with kanamycin at an OD_{600} of 0.1. The culture was incubated at 37 ºC with shaking at 200 RPM and protein expression was induced with 0.5 mM IPTG at an OD_{600} of 0.5. Cultures were grown overnight at 18 ºC with shaking at 200 RPM. Cells were harvested and the cell pellet resuspended in 50 mM Tris-HCl (pH 7.5), 250 mM NaCl, 20 mM imidazole (buffer A) supplemented with protease inhibitors (Roche). The cells were lysed by sonication and cell debris were eliminated by centrifugation (48,000 *x* g, 30 mins, 4 °C). His_6-Hcp1 was purified by immobilized metal affinity chromatography using nickel-Sepharose resin (GE Healthcare) equilibrated in buffer A and eluted off the resin with buffer A containing 500 mM instead of 20 mM imidazole. The protein was subsequently subjected to gel filtration chromatography in 50 mM Tris-HCl (pH 7.5), 250mM NaCl using a prepacked Superdex 75 10/300 GL size-exclusion column (GE Healthcare). Polyclonal antibody production was carried out by Eurogentec through their speedy 28-Day program (2 rabbits; 4 protein injections and 3 bleedings per rabbit).

Secretion assays. *P. putida* strains were grown in TSB for 8 hours at 30° C with shaking at 200 RPM and the extracellular fraction was obtained and analyzed as previously described (12). Briefly, the cell suspensions were spun three times at 10,000 *x g* for 20 minutes.

Bacterial pellets were normalized and added directly to 1x Laemmli buffer, whilst the culture supernatants were collected and precipitated with trichloroacetic (TCA) acid overnight. Precipitants were then washed with acetone and resuspended in 1x Laemmli buffer. All samples were boiled for 15 minutes. SDS-PAGE analysis was carried out using 8% or 15% BisTris NuPAGE gels (ThermoFisher Scientific), MES/SDS running buffer prepared according to the manufacturer's instructions and pre-stained protein markers (Kaleidoscope Prestained Standard, Bio-Rad). Proteins were transferred to Amersham Protran nitrocellulose membranes (0.45 µm pore size, GE Life Sciences) using a Trans-Blot Turbo transfer system (Bio-Rad) before blocking in 5% w/v skimmed milk/TBS-T and adding the primary and secondary antibodies. The following primary antibodies were used: rabbit anti-Hcp1 antibody (dilution 1:500 in 5 w/v % skimmed milk/TBS-T) and mouse *E. coli* anti-RNA polymerase beta (Neoclone) (dilution 1:5,000 in 5 w/v % skimmed milk/TBS-T). The following secondary antibodies were used: goat anti-rabbit IgG-HRP conjugate (Sigma Aldrich) (dilution 1:6,000 in 5% w/v skimmed milk/TBS-T) and goat anti-mouse IgG-HRP conjugate (Sigma Aldrich) (dilution 1:6,000 in 5% w/v skimmed milk/TBS-T). Membranes were washed three times for 5 minutes with TBS-T prior to development. HRP conjugates were visualized with the Luminata Forte Western HRP Substrate (Merck) using a Gel Doc XR+ Imager (Bio-Rad).

Bacterial two-hybrid assay. Protein-protein interactions were analyzed using the bacterial-two-hybrid (BTH) approach (13). Briefly, the proteins to be tested were independently fused to the T18 or T25 catalytic domains of the *B. pertussis* adenylate cyclase using plasmids pUT18 (or pUT18C) and pKT25, respectively. The two plasmids expressing the fusion proteins were simultaneously introduced into the reporter strain BTH101 by transformation and the plates were incubated at 30 °C for 24 hours. Three independent colonies for each plasmid combination were inoculated into 1 ml of LB (10 g/L NaCl) supplemented with ampicillin, kanamycin and 0.5 mM IPTG. After overnight growth at 30 °C with shaking at 200 RPM, 10 µl of each culture were spotted onto LB (10 g/L NaCl) agar (1.5% w/v) plates supplemented with ampicillin, kanamycin, 0.5 mM IPTG and 40 µg/ml X-Gal and incubated at 30 °C for 16 hours. At least three biologically independent experiments were performed for each protein pair.

Fluorescence microscopy. Fluorescence microscopy experiments were performed using sfGFP (14) and mScarlet-I (15) fusion proteins. The functionality of the T6SS in *P. putida rpoN* strains expressing fluorescent fusions of TssA1 and TagB1 from the native gene loci, was confirmed by performing secretion assays with the strains *P. putida rpoN* TssA1-sfGFP and *P. putida rpoN* TagB1-sfGFP, as described above (Fig. S14). Bacterial cultures intended for microscopy were grown in TSB supplemented with the appropriated antibiotics for at least 8 hours at 30ºC (*P. putida*) or 37 ºC (*P. aeruginosa*) with shaking at 200 RPM. Bacterial suspensions were then sub-cultured at an OD_{600} of 0.1 into 50 ml TSB and grown under the same conditions to an OD_{600} of 5 before visualization; *P. aeruginosa* PAO1 *rsmA tssB3::sfGfp* was grown at 25 °C with shaking at 200 RPM for 12 hours before visualization.

To visualize cells, 5 cm diameter glass bottom Petri dishes with a 3-cm diameter uncoated n° 1.5 glass window (MatTek Corporation) were used. 6 µl of bacterial culture were placed onto a 2.8 cm diameter (0.5 cm thickness) circular slab of Vogel-Bonner salts agar (2% w/v) which was, in turn, placed face down onto the glass bottom window of the Petri dish, so that the cells were sandwiched between the glass and the solid medium. Samples were immediately transferred to the microscope and imaged using an Axio Observer Z1 (Zeiss) inverted widefield microscope equipped with a Plan-Apochromat 63x/1.4 NA Oil Ph3 M27

objective (Zeiss), a SpectraX LED light engine (Lumencore), an ORCA-Flash 4.0 digital CMOS camera (Hamamatsu) and an environmental control system. Images were acquired using 1x1 binning, corresponding to a pixel size of 0.103 x 0.103 μm. All microscopy was performed at the Facility for Imaging by Light Microscopy (FILM) at Imperial College London, UK.
For the visualization of *P. putida* strains expressing TagB1-sfGFP or TssB1-sfGFP, fluorescence images were acquired every 2 s for a total of 180 s using an exposure time of 195 ms. For *P. putida* simultaneously expressing TssB1-mScarlet-I and sfGFP-TssA1, fluorescence images were acquired every 4 s for a total of 180 s using an exposure time of 500 ms for sfGFP and 250 ms for mScarlet-I. For *P. putida* simultaneously expressing TssB1-mScarlet-I and TagB1-sfGFP, fluorescence images were acquired every 2 s for a total of 180 s using an exposure time of 200 ms for sfGFP and 350 ms for mScarlet-I. For the visualization of *P. aeruginosa* expressing TssB1-sfGFP, fluorescence images were acquired every 2 s for a total of 180 s using an exposure time of 80 ms. For *P. aeruginosa* expressing TssB2-sfGFP, fluorescence images were acquired every 20 s for a total of 1200 s using an exposure time of 80 ms; the microscope chamber was set to 30 °C and the Definite Focus autofocus system was used. For *P. aeruginosa* expressing TssB3-sfGFP, fluorescence images were acquired every 30 s for a total of 450 s using an exposure time of 80 ms. Finally, for *P. aeruginosa* expressing TagJ1-sfGFP, fluorescence images were acquired every 60 s for 300 s using an exposure time of 400 ms.

Image analysis and presentation. Image processing and analysis was performed using FIJI (16) in conjunction with the MicrobeJ (17) plugin. Generation of images was performed using the ZEN Blue software (Zeiss) and FIJI. Prior to analysis, x-y drift present in time-lapse series was corrected for each channel using the StackReg plug-in for FIJI and the "rigid body" transformation setting (18). Photobleaching in time-lapse series was corrected using the "simple ratio" bleach correction method within FIJI. Semi-automatic particle detection, segmentation and generation of cell masks were performed on phase-contrast images using the "medial axis" cell contour method within MicrobeJ; all cell masks were manually checked before further analysis. Cell counts and cell width data were directly extracted from manually curated cell masks generated using this method. Detection of fluorescent *P. putida* TagB1 foci was performed on fluorescence images using the local maxima detection algorithm implemented in FIJI, in conjunction with the "foci" particle conversion method implemented in MicrobeJ. Changes in TagB1 foci intensity over time were quantified from time-lapse series by determining the mean pixel intensity values of regions of interest (ROIs) using the "Plot Z-axis profile" tool within FIJI. Data are presented normalized to the average fluorescence intensity of the cytosol of three cells (from the same time-lapse series) in which no TagB1 foci were detected during the course of imaging. Enumeration, localization and sheath length analysis of TssB1-sfGFP sheaths was performed manually for all visualized strains. Sheath length measurements were performed on time-lapse series acquired using *P. putida* strains expressing TssB1-sfGFP; measurements were performed on the frame directly prior to that in which sheath contraction was observed, using a custom line/ROI measurement macro in FIJI. Time to sheath contraction data were extracted manually from time-lapse series covering full sheath polymerization and contraction events. For image presentation, frames of interest were extracted from time-lapse series, corrected as described above, and scale bars created using the Scale Bar function in FIJI. Videos were prepared from the same corrected images and encoded using QuickTime (H.264 codec) (Apple Inc.). All videos are presented at 7 frames/s as .mov files.

Statistical analysis of experimental data. Unless otherwise stated, statistical analysis was performed in GraphPad PRISM v8.0.2 using an unpaired two-tailed T-test with Welch's correction, multiple unpaired T-tests corrected for multiple comparisons using the Holm-Sidak method, or a one-way ANOVA with Tukey's multiple comparisons test, as appropriate. Statistical significance was defined as $p < 0.05$. Detailed information for each figure is provided below, and is also included in the relevant figure legends:

Fig. 2G: unpaired T-test with Welch's correction; *P. putida rpoN* TssB1-sfGFP (n=98), *P. putida rpoN tagB1* TssB1-sfGFP (n=95); 174.8 degrees of freedom, t-value=16.71; p=5.065 x 10^{-38} (significance).
Fig. 2H: unpaired T-test with Welch's correction; *P. putida rpoN* TssB1-sfGFP (n=706), *P. putida rpoN tagB1* TssB1-sfGFP (n=693); 1389 degrees of freedom, t-value=0.2638; p=0.7920 (non-significance).
Fig. 3D: unpaired T-tests corrected for multiple testing using the Holm-Sidak method. For *E. coli* competitions n=5; *P. putida* vs. *P. putida tssA1* adjusted p=0.001167 (8 degrees of freedom), *P. putida* vs. *P. putida tagB1* adjusted p=0.019402 (24 degrees of freedom), *P. putida tssA1* vs. *P. putida tagB1* adjusted p=0.030542 (24 degrees of freedom) (all significant). For *X. campestris* competitions n=3; *P. putida* vs. *P. putida tssA1* adjusted p=0.009447 (4 degrees of freedom), *P. putida* vs. *P. putida tagB1* adjusted p=0.019402 (24 degrees of freedom), *P. putida tssA1* vs. *P. putida tagB1* adjusted p=0.001820 (24 degrees of freedom) (all significant). For *A. tumefaciens* competitions n=3; *P. putida* vs. *P. putida tssA1* adjusted p=0.006286 (4 degrees of freedom), *P. putida* vs. *P. putida tagB1* adjusted p=0.019402 (24 degrees of freedom), *P. putida tssA1* vs. *P. putida tagB1* adjusted p=0.030542 (24 degrees of freedom) (all significant). For *P. syringae* competitions n=3; *P. putida* vs. *P. putida tssA1* adjusted p=0.006286 (4 degrees of freedom), *P. putida* vs. *P. putida tagB1* adjusted p=0.000135 (24 degrees of freedom), *P. putida tssA1* vs. *P. putida tagB1* adjusted $p < 0.000001$ (24 degrees of freedom) (all significant). For *P. savastanoi* competitions n=3; *P. putida* vs. *P. putida tssA1* adjusted p=0.006286 (4 degrees of freedom), *P. putida* vs. *P. putida tagB1* adjusted p=0.000069 (24 degrees of freedom), *P. putida tssA1* vs. *P. putida tagB1* adjusted p=0.000409 (24 degrees of freedom) (all significant).
Fig. 3E: one-way ANOVA with Tukey's multiple comparison test; n=4; 11 degrees of freedom; F value=73.55; $p < 0.0001$ (significant). *P. putida* vs. *P. putida tssA1* adjusted $p < 0.0001$, *P. putida* vs. *P. putida tagB1* adjusted $p < 0.0001$, *P. putida tssA1* vs. *P. putida tagB1* adjusted p=0.0051 (all significant).

In silico analyses. In order to categorize TssA-like proteins and identify TssA-related accessory components, an *in silico* study of 100 T6SS clusters was performed. Proteins encoding the core components and accessory proteins of the selected T6SS clusters were downloaded from the SecReT6 website (19) and organized by phylogenetic group (groups 1-5, with phylogenetic subgroups 4A and 4B being considered separately) (20); 20 T6SS clusters per phylogenetic group were examined. The T6SS cluster that is best characterized from each phylogenetic group was set as the reference cluster for the analysis (distinct clusters from *P. aeruginosa*, *E. coli*, *P. putida* and *A. tumefaciens* were selected as references). The reference clusters were used as queries in a blastp search (21) against the remaining selected T6SS clusters from the same phylogenetic group. The percentage identity and coverage resulting from the search can be found in Dataset S1. Through this analysis, five families of TssA-like proteins were identified, and their domains were further characterized using the "NCBI Conserved Domain" function and the SMART website (22).

Phylogenetic analyses for TssA and Hcp proteins were performed as previously described (23) using MEGA7 (24). Briefly, for each protein, a sequence alignment was carried out using the ClustalW algorithm, and subsequently a maximum-likelihood tree with 500 bootstrap replicates was built. The online Interactive Tree Of Life (iTOL) tool (25) was used for display and annotation of the resulting phylogenetic trees. Phylogenetic analysis of TssA was performed using all sequences of short TssA proteins listed in Dataset S1, whilst for Hcp the six and five annotated Hcp proteins from *P. putida* and *P.* aeruginosa, respectively were used.

Data availability. All data generated during this study that support the findings are included in the manuscript or in the SI Appendix.

SUPPLEMENTARY INFORMATION FIGURES

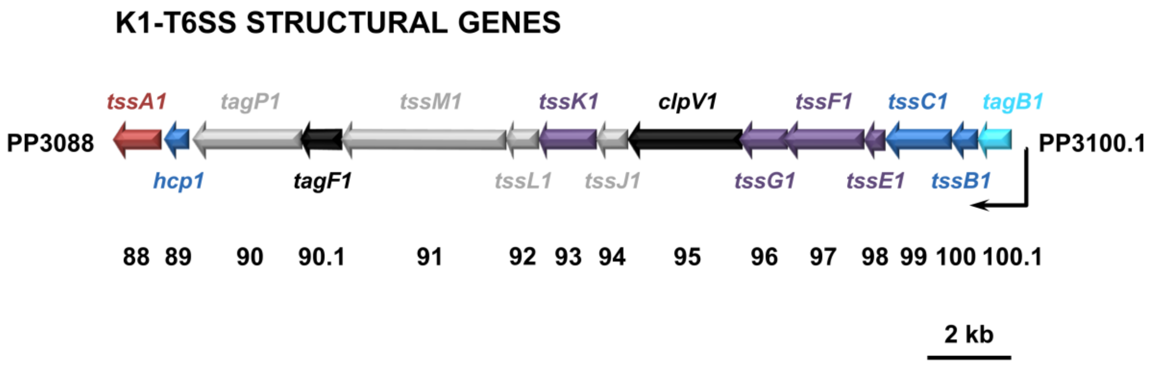

Fig. S1. Schematic representation of the genomic organization of the structural genes of the K1-T6SS cluster from *P. putida* KT2440. Genes encoding membrane complex proteins are shown in grey, whereas baseplate and tail components are depicted in purple and blue, respectively. *tssA1* is in red, *tagB1* in cyan and *tagF1* and *clpV1* are colored black. *vgrG1* is not shown, as it is located downstream in the effector module (26), which is omitted from this schematic.

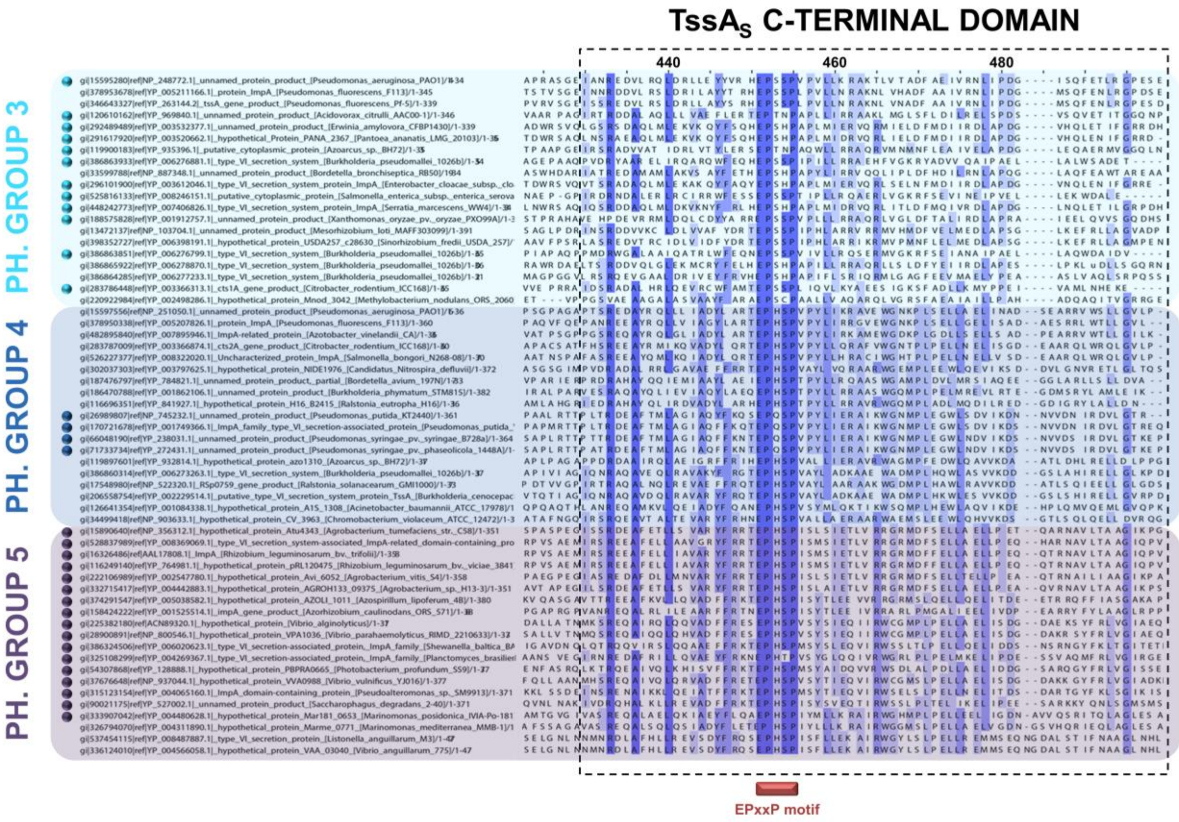

Fig. S2. Sequence alignment of the C-terminal domains of the 60 short TssA proteins included in Dataset S1. Aligned proteins have a conserved N-terminal ImpA domain, which is a hallmark of the TssA protein family (27, 28). The distinct C-terminal domain (27) of the TssA$_S$ proteins depicted here is also largely conserved, regardless of whether the corresponding T6SS contains a TagB or TagJ component; presence of *tagB* or *tagJ* in the T6SS cluster is indicated by a circle. The highly conserved EPxxP motif of the TssA$_S$ C-terminal domain is marked by a red bar.

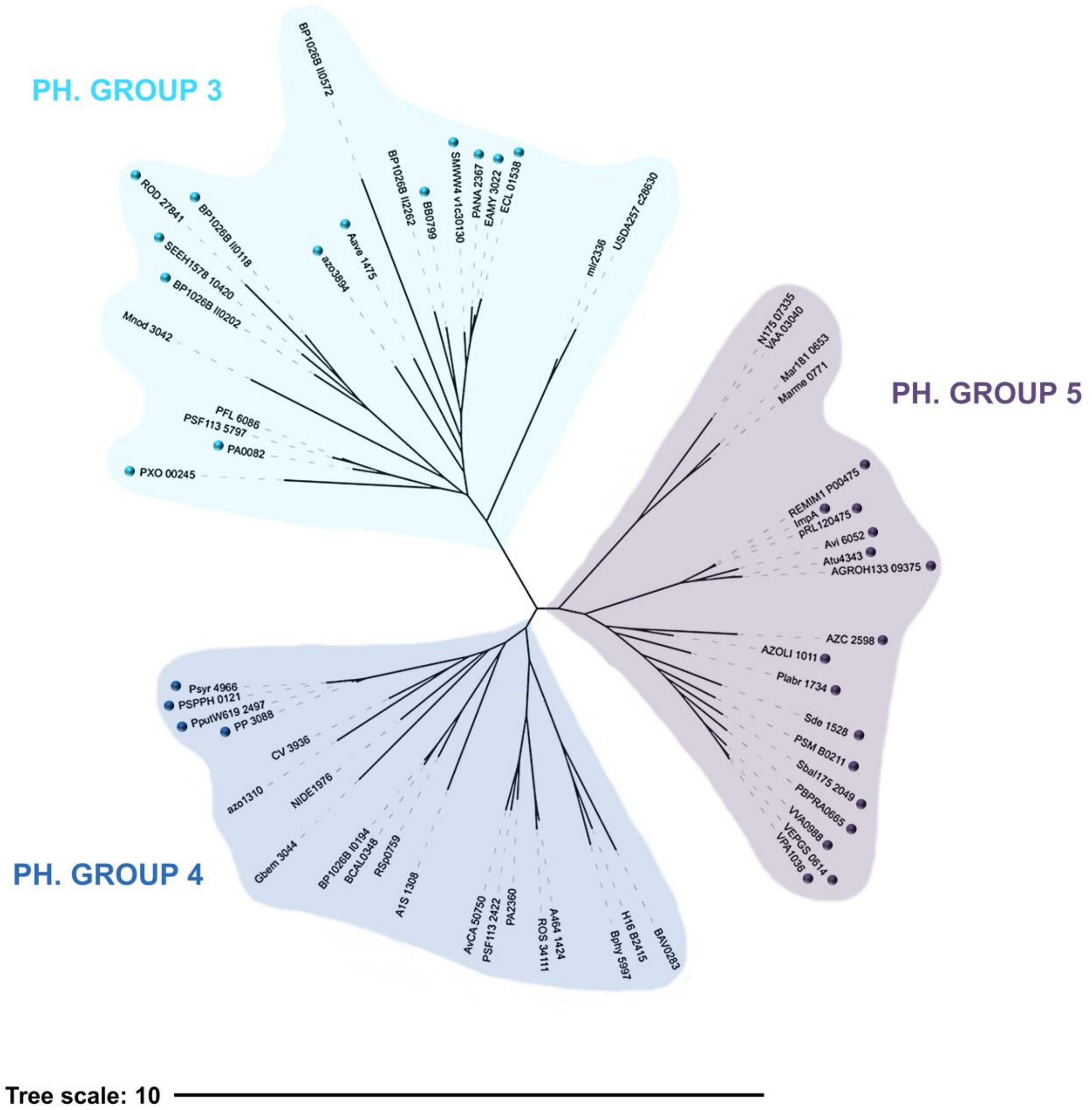

Fig. S3. Phylogenetic distribution of short TssA proteins. Maximum-likelihood tree for $TssA_S$ proteins built with MEGA7 and displayed using iTOL. $TssA_S$ proteins cluster in the same phylogenetic groups as the corresponding TssB proteins, which are commonly used when T6SS phylogeny is examined (20). Phylogenetic clustering is based on the analysis of Boyer et al. (20). The presence of *tagB* or *tagJ* in the corresponding T6SS cluster is indicated by a circle.

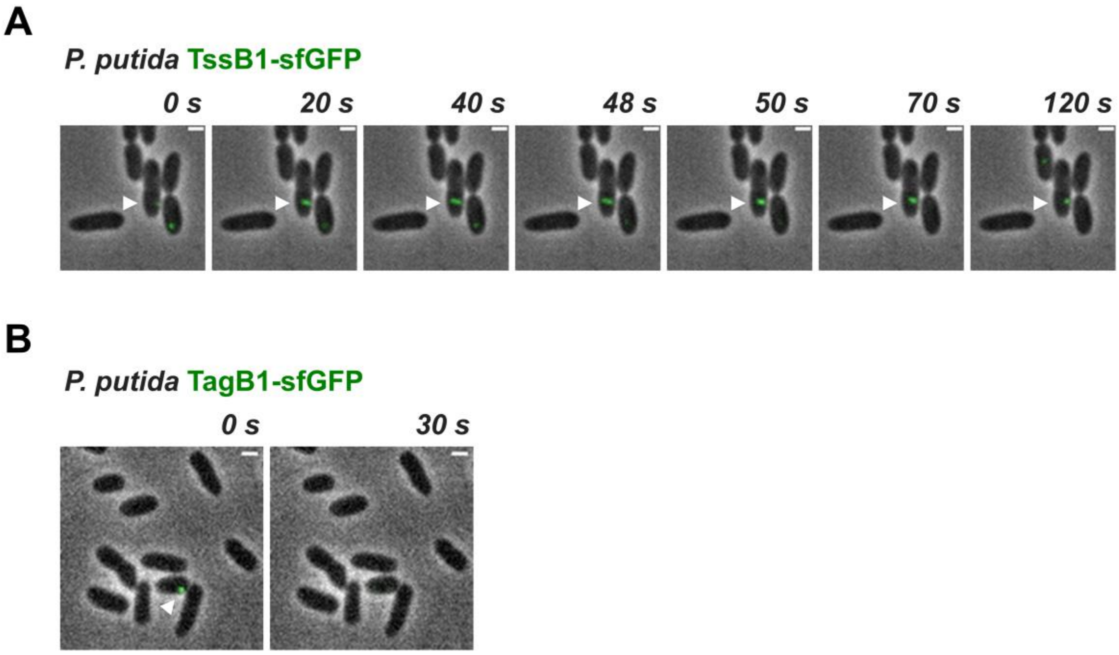

Fig. S4. TssB1-sfGFP and TagB1-sfGFP show the same *in vivo* behavior in a wild-type *P. putida* strain as in *P. putida rpoN*. (A) *In vivo* imaging of *P. putida* TssB1-sfGFP. The full cycle of T6SS assembly over approximately 120 seconds is shown for a representative sheath (white arrowhead). The presented panels are selected images from a fluorescence microscopy time-lapse recording of *P. putida* expressing TssB1-sfGFP from the native *tssB1* locus; images were recorded every 2 s. **(B)** *In vivo* imaging of *P. putida* TagB1-sfGFP. Over time, TagB1 forms transient foci which abruptly disappear (white arrowhead). The presented panels are selected images from a fluorescence microscopy time-lapse recording of *P. putida* expressing TagB1-sfGFP from the native *tagB1* locus. For (A) and (B) scale bars represent 1 μm.

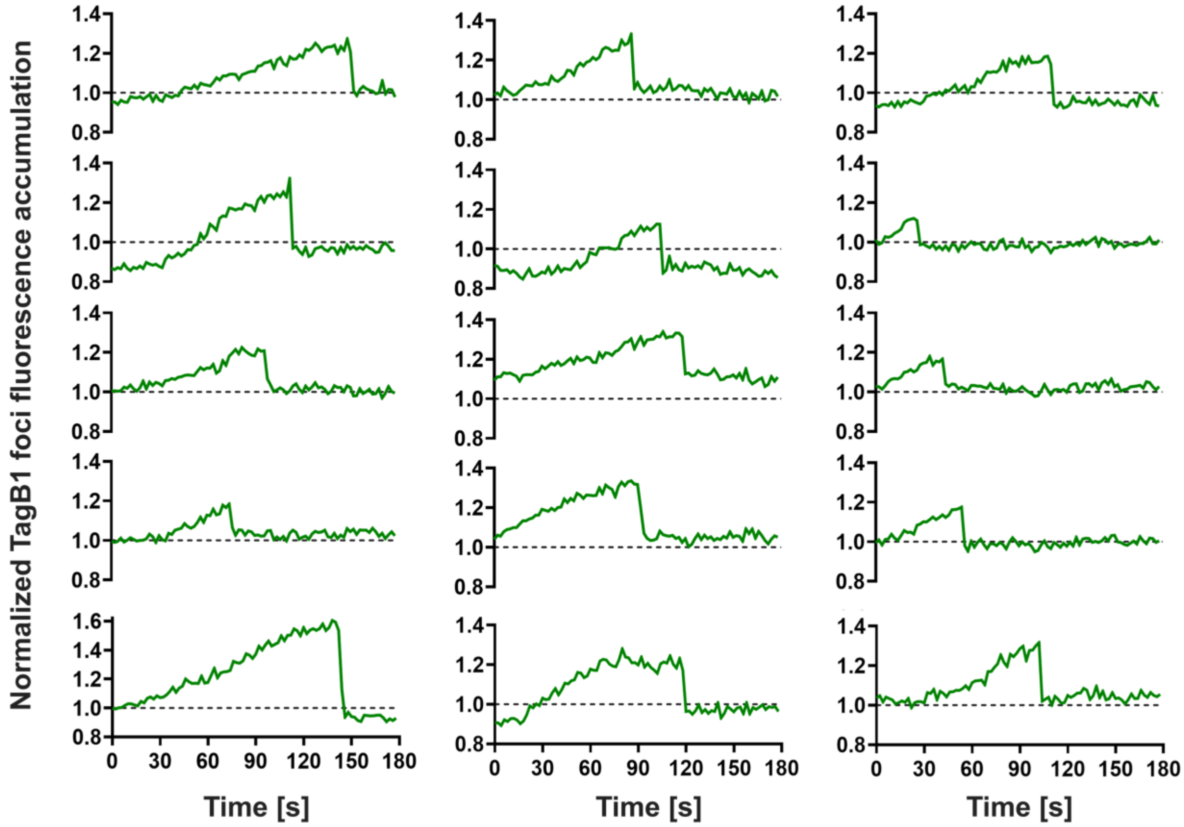

Fig. S5. Quantification of fluorescence intensity demonstrates that TagB1 foci accumulate over time before abruptly disappearing. Further to the five foci shown in Fig. 2B, 15 additional representative foci were analyzed. The dotted line indicates the average signal in areas of the cytosol where no foci were detected. The data were generated from fluorescence microscopy time-lapse recordings of *P. putida rpoN* expressing TagB1-sfGFP from the native *tagB1* locus (see also Video S2); images were recorded every 2 s.

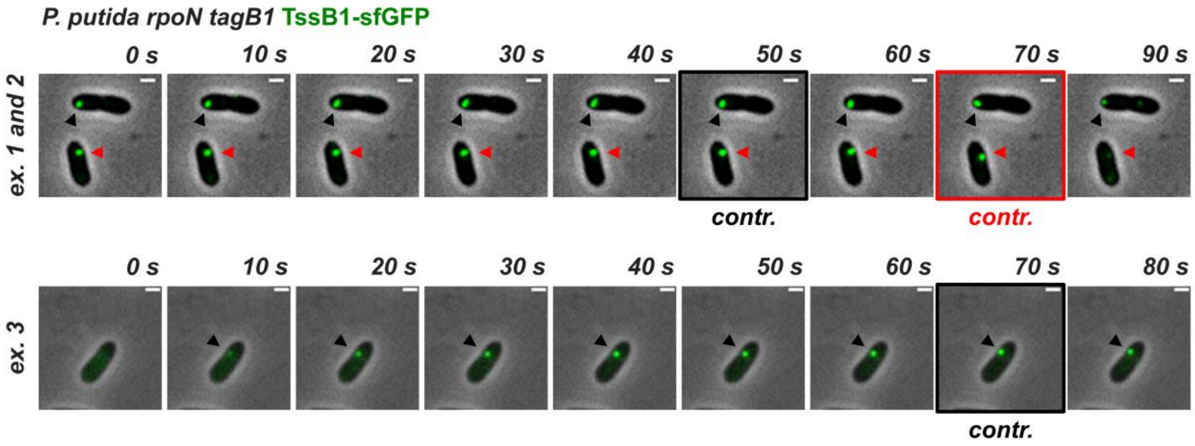

Fig. S6. Absence of TagB1 prevents full extension of the K1-T6SS sheath. *In vivo* imaging of *P. putida rpoN tagB1* TssB1-sfGFP. Three examples ("ex.") of T6SS sheaths that contract before fully extending to the opposite side of the cell are shown. Black or red arrowheads indicate the sheaths of interest and panels showing sheath contraction ("contr.") are marked with a black or red outline, respectively. The presented panels are selected images from two fluorescence microscopy time-lapse recordings of *P. putida rpoN* expressing TssB1-sfGFP from the native *tssB1* locus. Images were recorded every 2 s and scale bars represent 1 μm. An image from example 2 (top row; red arrowhead) was used to showcase the *P. putida rpoN tagB1* TssB1-sfGFP behavior in Fig. 2F.

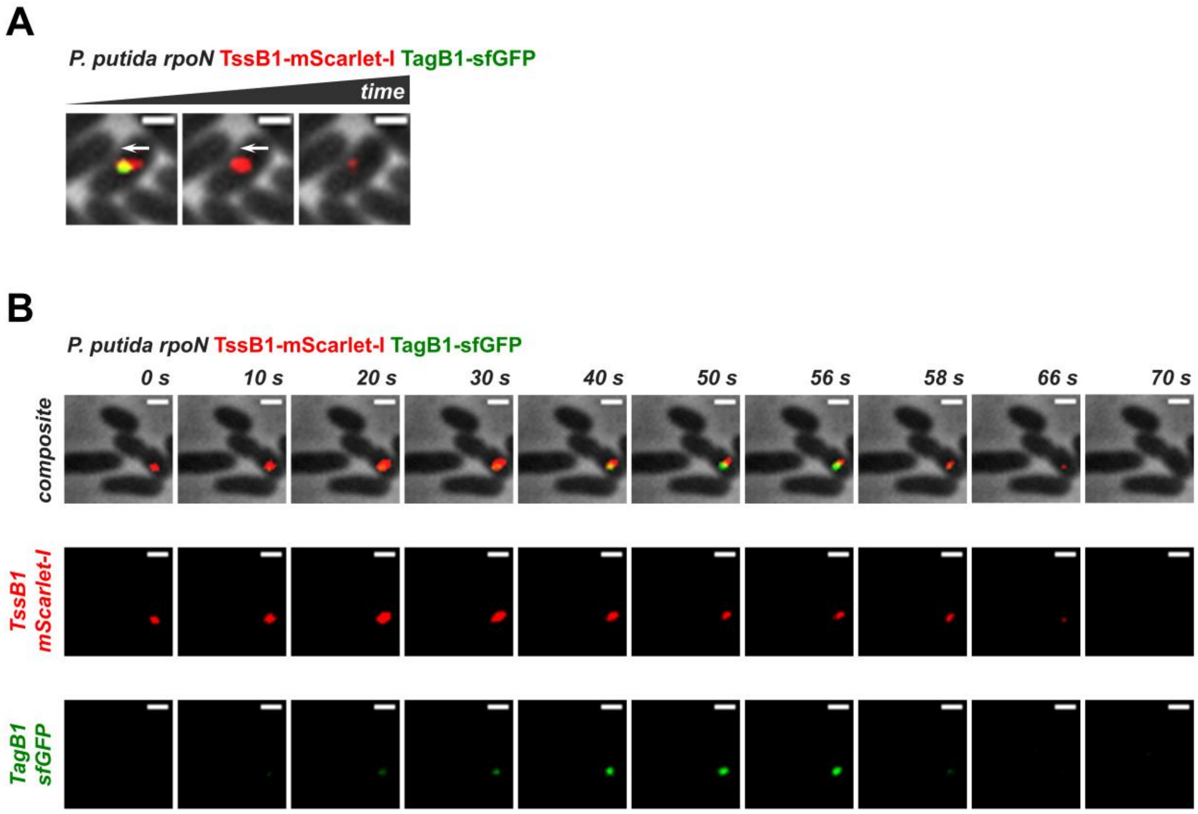

Fig. S7. *P. putida* TagB1 localizes to the baseplate. The presented panels are selected images from a fluorescence microscopy time-lapse recording of *P. putida rpoN* expressing TagB1-sfGFP from the native *tagB1* locus. The gene encoding TssB1-mScarlet-I was integrated into the chromosome whilst the native *tssB1* copy was still present. Scale bars represent 1 µm. **(A)** TagB1-sfGFP foci are associated with the end of the extended sheath towards which contraction occurs. The white arrow indicates the direction of sheath contraction. **(B)** Images used to generate Fig. 3B. Phase-contrast composite images displaying both fluorescence channels are shown in the top row, and images from individual fluorescence channels are shown in the middle and bottom rows (mScarlet-I and sfGFP, respectively).

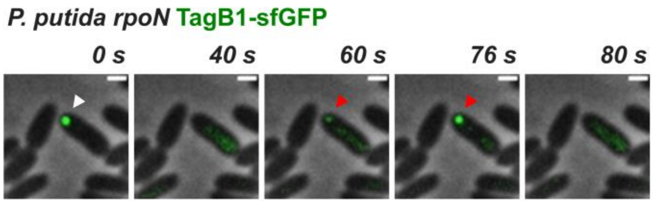

Fig. S8. ***P. putida*** **TagB1-sfGFP foci re-appear in the same location after a contraction event.** A TagB1-sfGFP focus (white arrowhead) disappears, presumably after sheath contraction, and a new focus appears (red arrowhead) in exactly the same location. The presented panels are selected images from a fluorescence microscopy time-lapse recording of *P. putida rpoN* expressing TagB1-sfGFP from the native *tagB1* locus. Images were recorded every 2 s and scale bars represent 1 µm.

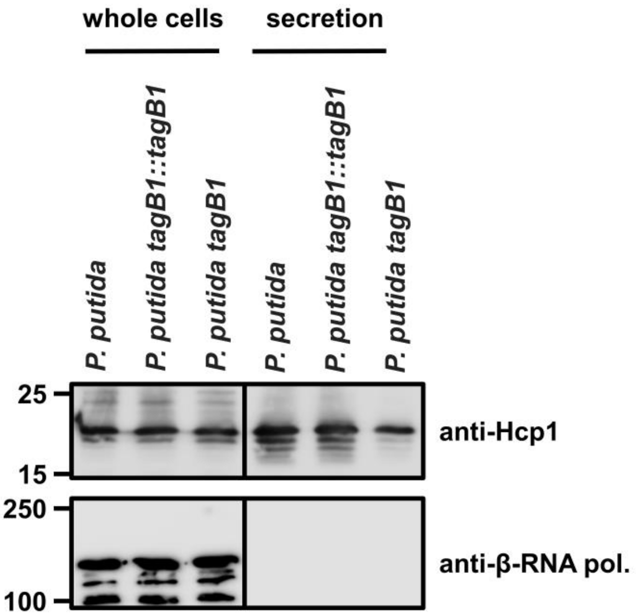

Fig. S9. Re-introduction of *tagB1* in its native locus in *P. putida tagB1* (strain *P. putida tagB1::tagB1*) restores Hcp1 secretion to wild-type levels. Hcp1 expression and presence in the culture supernatant were assessed for *P. putida* strains. Positions of molecular weight markers are shown on the left, the β-subunit of the *E. coli* RNA polymerase (β-RNA pol.) was used as a loading and bacterial lysis control and black lines indicate where the membrane was cut. A representative blot from three independent experiments is presented.

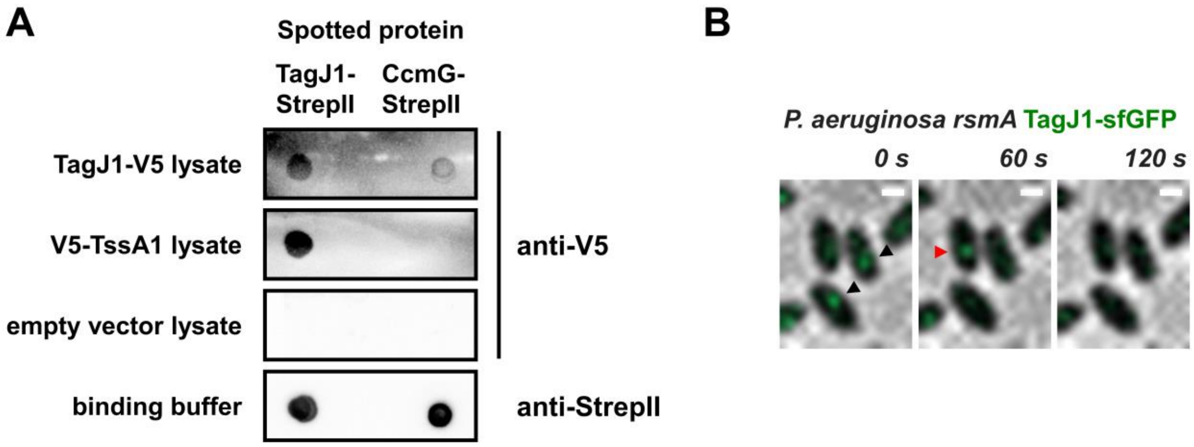

Fig. S10. *P. aeruginosa* TagJ1 exhibits similar properties and *in vivo* behavior as *P. putida* TagB1. (A) Dot blot assays showing interaction of *P. aeruginosa* TagJ1 with itself and its cognate TssA1. CcmG-StrepII was used as a binding control protein; similar amounts of pure TagJ1-StrepII and CcmG-StrepII were spotted on the membrane. **(B)** *In vivo* imaging of *P. aeruginosa* TagJ1-sfGFP. TagJ1 forms transient foci which abruptly disappear (black arrowheads). Disappearance of foci is not due to photobleaching as new foci appear in directly adjacent cells (red arrowhead). The presented panels are selected images from a fluorescence microscopy time-lapse recording of *P. aeruginosa rsmA* expressing TagJ1-sfGFP from the native *tagJ1* locus. Images were recorded every 2 s and scale bars represent 1 μm.

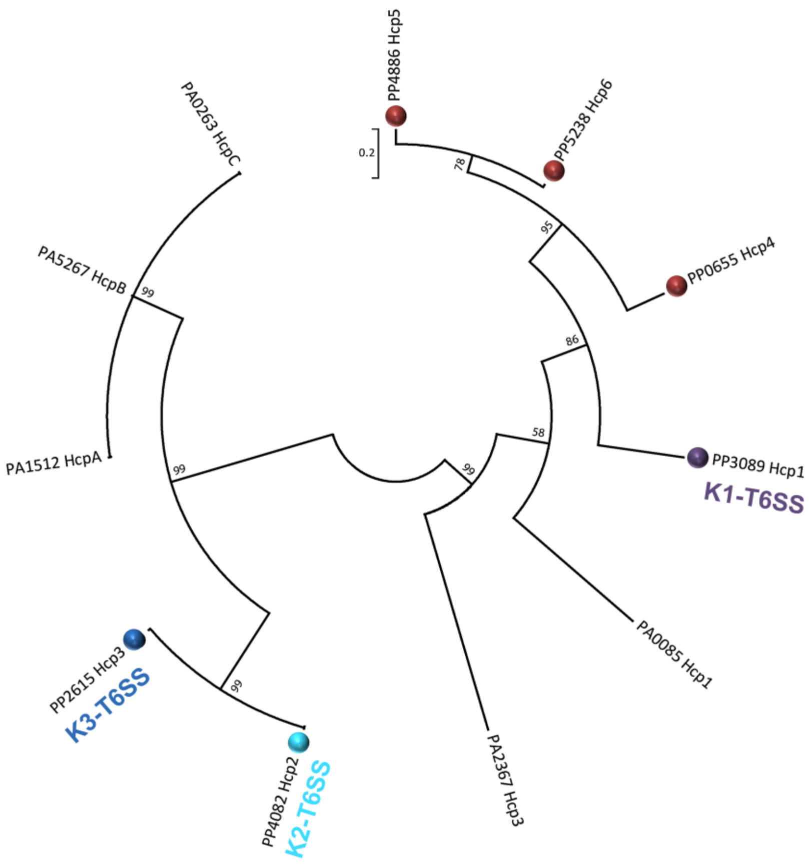

Fig. S11. Phylogenetic distribution of Hcp proteins in *Pseudomonas* species. Maximum-likelihood tree of *P. putida* and *P. aeruginosa* Hcp proteins built with MEGA7 and displayed using iTOL. *P. putida* orphan Hcp proteins, Hcp4, Hcp5 and Hcp6 (marked with red circles) (26) cluster with Hcp1 from the K1-T6SS (marked with a purple circle). By contrast, *P. putida* Hcp2 (cyan circle) and Hcp3 (blue circle), belonging to the K2-T6SS and K3-T6SS, respectively, are distinct from Hcp1 and cluster with *P. aeruginosa* Hcp proteins.

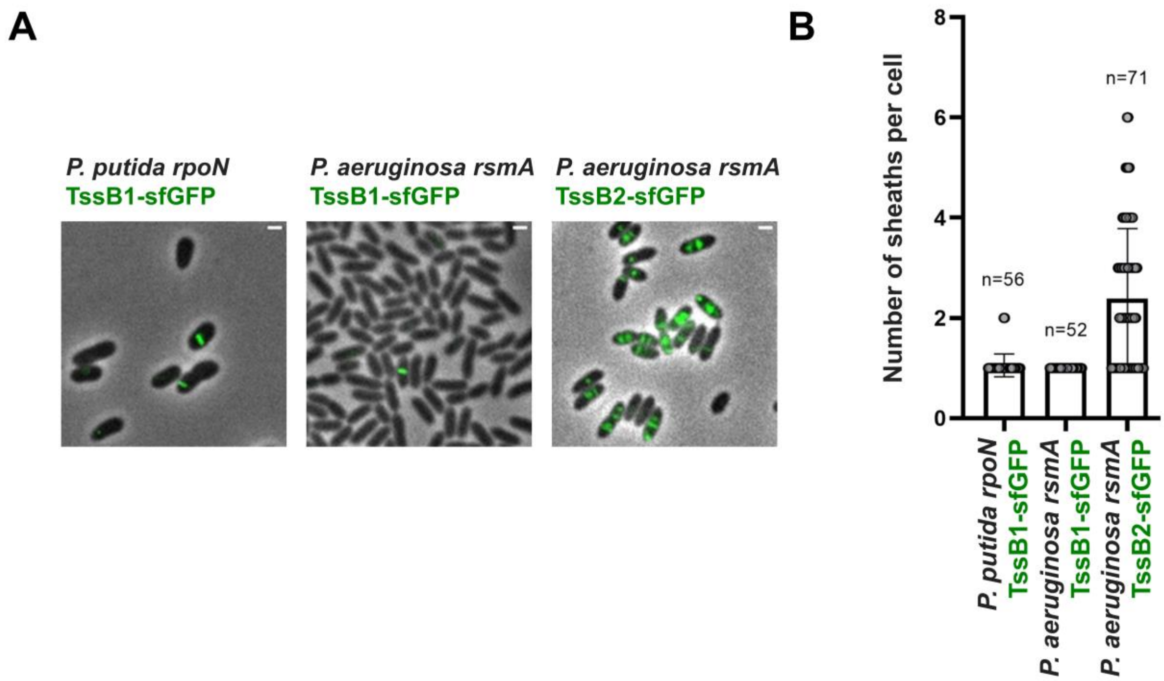

Fig. S12. Distinct anchoring modes result in different numbers of T6SS apparatuses per cell. (A) Representative fluorescence microscopy images from a fluorescence microscopy time-lapse recording of *P. putida rpoN* expressing TssB1-sfGFP from the native *tssB1* locus (left), and *P. aeruginosa rsmA* expressing TssB1-sfGFP (middle) and TssB2-sfGFP (right) from the native *tssB1* and *tssB2* loci, respectively. Scale bars represent 1 µm. **(B)** Quantification of (A) for a specific time point during the time lapse recording. One apparatus is usually present at any given time for the *P. putida* K1-T6SS and *P. aeruginosa* H1-T6SS, but up to six sheaths can be simultaneously detected for the *P. aeruginosa* H2-T6SS. n indicates the number of cells included in the analysis.

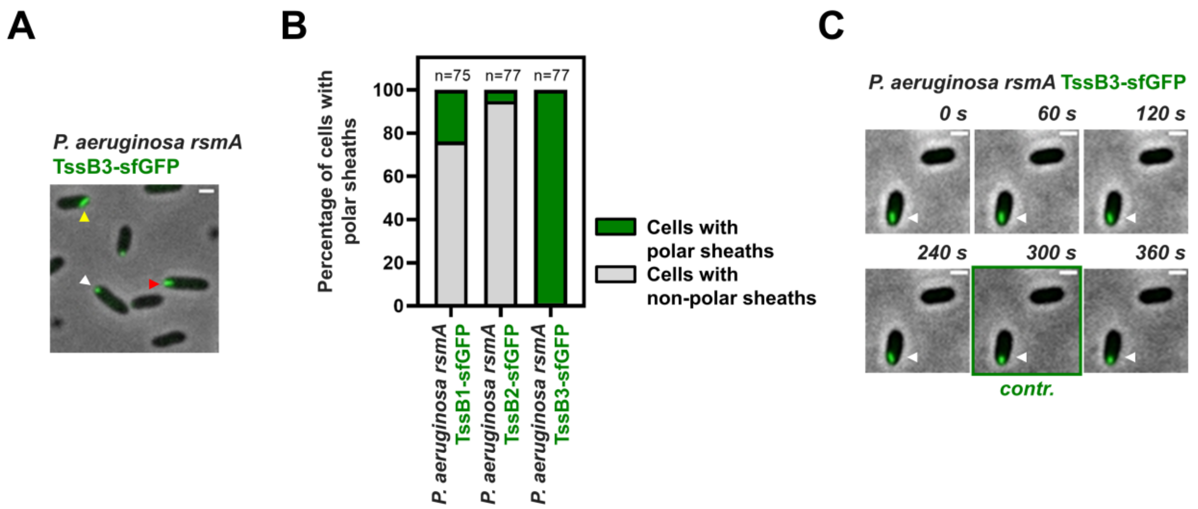

Fig. S13. The *P. aeruginosa* H3-T6SS is not stabilized in a classical manner. (A) Representative fluorescence microscopy image of *P. aeruginosa rsmA* expressing TssB3-sfGFP from the native *tssB3* locus. TssB3-sfGFP foci (white arrowhead) and H3-T6SS sheaths (red and yellow arrowheads) are exclusively localized at the cell pole. Sheath structures grow from the pole either horizontally without reaching the opposite side of the cell (red arrowhead) or diagonally eventually leaning against the side of the cell (yellow arrowhead). Scale bars represent 1 µm. **(B)** Quantification of sheaths with polar localization in the *P. aeruginosa* H1-, H2- and H3-T6SS. n indicates the number of cells included in the analysis. **(C)** *P. aeruginosa* H3-T6SS sheaths contract. A “floating” polar H3-T6SS sheath (white arrowhead) contracts over time; contraction (“contr.”) is marked with a green panel outline. The presented panels are selected images from a fluorescence microscopy time-lapse recording of *P. aeruginosa rsmA* expressing TssB3-sfGFP from the native *tssB3* locus. Images were recorded every 30 s and scale bars represent 1 µm.

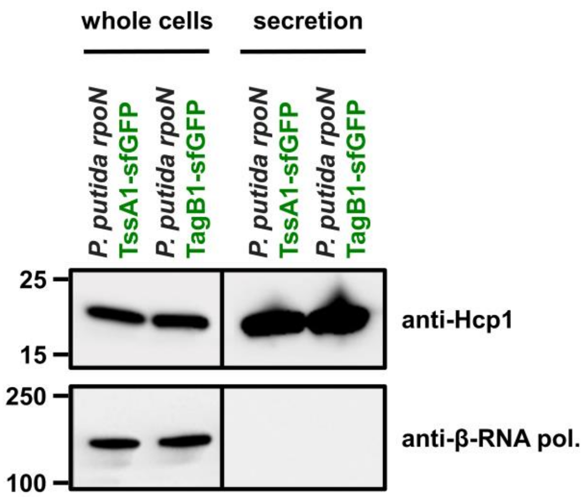

Fig. S14. The functionality of the T6SS in *P. putida rpoN* strains expressing fluorescent fusions of TssA1 and TagB1 was confirmed by monitoring Hcp1 secretion. Hcp1 expression and presence in the culture supernatant were assessed for *P. putida rpoN* strains expressing TssA1-sfGFP and TagB1-sfGFP from the native gene loci. Positions of molecular weight markers are shown on the left, the β-subunit of the *E. coli* RNA polymerase (β-RNA pol.) was used as a loading and bacterial lysis control and black lines indicate where the membrane was cut. A representative blot from three independent experiments is presented.

SUPPLEMENTARY INFORMATION TABLES

Table S1. Bacterial strains used in this study.

Name	Description	Source
Escherichia coli		
DH5α	F^- *end*A1 *gln*V44 *thi*-1 *rec*A1 *rel*A1 *gyr*A96 *deo*R *nup*G *pur*B20 φ80d*lac*ZΔM15 Δ(*lac*ZYA-*arg*F)U169 *hsd*R17($r_K^- m_K^+$) $λ^-$	(29)
CC118λpir	*ara*D Δ(*ara*, *leu*) Δ*lac*Z74 *pho*A20 *gal*K *thi*-1 *rsp*E *rpo*B *arg*E *rec*A1 λ*pir*	(30)
HB101	*sup*E44 *hsd*S20 *rec*A13 *ara*-14 *pro*A2 *lac*Y1 *gal*K2 *rps*L20 *xyl*-5 *mtl*-1	(31)
BL21(DE3)	F^- *omp*T *gal dcm lon hsd*S_B($r_B^- m_B^-$) λ(DE3 [*lac*I *lac*UV5-T7p07 *ind*1 *sam*7 *nin*5]) [*mal*B^+]$_{K-12}$($λ^S$)	(32)
BTH101	F^- *cya*-99 *ara*D139 *gal*E15 *gal*K16 *rps*L1(Str^R) *hsd*R2 *mcr*A1 *mcr*B1	(13)
Pseudomonas putida		
KT2440R	Rif^R	(33)
KT2440R *tssA1*	Markerless mutant, Rif^R	(26)
KT2440R *hcp1*	Markerless mutant, Rif^R	This study
KT2440R *tagB1*	Markerless mutant, Rif^R	This study
KT2440R *tagB1::tagB1*	Markerless mutant, Rif^R	This study
KT2440R *tagB1::sfgfp*	Rif^R	This study
KT2440R *tssB1*::*sfgfp*	Rif^R	This study
KT2440 *rpoN*	*rpoN*::*aphA*, Kan^R	(34)
KT2440 *rpoN tssA1*	*rpoN*::*aphA*, Kan^R	(26)
KT2440 *rpoN tssA1::sfgfp*	*rpoN*::*aphA*, Kan^R	This study
KT2440 *rpoN tssA1 tssB1*::*sfgfp*	*rpoN*::*aphA*, Kan^R	This study
KT2440 *rpoN tssA1 tagB1*::*sfgfp*	*rpoN*::*aphA*, Kan^R	This study
KT2440 *rpoN tagB1*	*rpoN*::*aphA*, Kan^R	This study
KT2440 *rpoN tagB1::sfgfp*	*rpoN*::*aphA*, Kan^R	This study
KT2440 *rpoN tagB1::sfgfp tssB1-mScarlet-I*	*rpoN*::*aphA*, Kan^R, Tet^R	This study
KT2440 *rpoN tagB1 tssB1*::*sfgfp*	*rpoN*::*aphA*, Kan^R	This study
KT2440 *rpoN tssB1*::*sfgfp*	*rpoN*::*aphA*, Kan^R	This study
KT2440 *rpoN tssB1*::*mScarlet-I*	*rpoN*::*aphA*, Kan^R	This study
KT2440 *rpoN tssB1*::*mScarlet-I tssA1::sfgfp*	*rpoN*::*aphA*, Kan^R	This study
KT2440 *rpoN tagB1::(StrepII)*$_2$	*rpoN*::*aphA*, Kan^R	This study
Pseudomonas aeruginosa		
PAO1 *rsmA*	Markerless mutant	(35)
PAO1 *rsmA tssB1*::*sfgfp*	Markerless mutant	This study
PAO1 *rsmA tagJ1*::*sfgfp*	Markerless mutant	This study
PAO1 *rsmA tssB2*::*sfgfp*	Markerless mutant	This study
PAO1 *rsmA tssB3*::*sfgfp*	Markerless mutant	This study
**Plant pathogens**		
Xanthomonas campestris pv. campestris IVIA 2734-1	-	(26)
Agrobacterium tumefaciens C58	-	(36)
Pseudomonas syringae pv tomato DC3000	-	(37)
Pseudomonas savastanoi pv savastanoi strain NCPPB 3335	-	(38)

Table S2. Plasmids used in this study. Superscripts “*PP*” and “*PA*” indicate *P. putida* and *P. aeruginosa* components (genes or proteins), respectively; these abbreviations are solely used in this table for brevity.

Name	Description	Source
pCR-BluntII-TOPO	Cloning vector, ColE1 *ori*, Kan^R	Invitrogen
pETDuet-1	Dual expression vector, ColE1 *ori*, T7 promoter, MCS1 and MCS2, Amp^R	Novagen
pET28a	Expression vector, pBR322 *ori*, T7 promoter, MCS, Kan^R	Novagen
pET28a-His-*hcp1*^{PP}	*hcp1*^{PP} encoding Hcp1^{PP} with an N-terminal His_6 tag cloned into pET28a, Kan^R	This study
pETDuet-1-His-*tssA1*^{PP}	*tssA1*^{PP} encoding TssA1^{PP} with an N-terminal His_6 tag cloned into pETDuet-1 MCS1, Amp^R	This study
pETDuet-1-V5-*tssA1*^{PP}	*tssA1*^{PP} encoding TssA1^{PP} with a N-terminal V5 tag cloned into pETDuet-1 MCS1, Amp^R	This study
pETDuet-1-*tagB1*^{PP}-StrepII	*tagB1*^{PP} encoding TagB1^{PP} with a C-terminal StrepII tag cloned into pETDuet-1 MCS2, Amp^R	This study
pETDuet-1-*tagB1*^{PP}-V5	*tagB1*^{PP} encoding TagB1^{PP} with a C-terminal V5 tag cloned into pETDuet-1 MCS2, Amp^R	This study
pETDuet-1-His-*tssA1*^{PA}	*tssA1*^{PA} encoding TssA1^{PA} with an N-terminal His_6 tag cloned into pETDuet-1 MCS1, Amp^R	This study
pETDuet-1-V5-*tssA1*^{PA}	*tssA1*^{PA} encoding TssA1^{PA} with an N-terminal V5 tag cloned into pETDuet-1 MCS1, Amp^R	This study
pETDuet-1-*tagJ1*^{PA}-StrepII	*tagJ1*^{PA} encoding TagJ1^{PA} with a C-terminal StrepII tag cloned into pETDuet-1 MCS2, Amp^R	This study
pETDuet-1-*tagJ1*^{PA}-V5	*tagJ1*^{PA} encoding TagJ1^{PA} with a C-terminal V5 tag cloned into pETDuet-1 MCS2, Amp^R	This study
pCcmG1	L36-A184 of *Escherichia coli* CcmG expressed from pET22b(+), Amp^R	Mavridou lab
mini-CTX1-P*lac*	Plasmid for the integration of genes into the *att* site of pseudomonal chromosomes, pMB1-derived *ori*, P*lac*, Tet^R	(5)
mini-CTX1-P*lac-tssB1*^{PP}-*mScarlet-I*	*tssB1*^{PP} encoding TssB1^{PP} C-terminally fused to m-Scarlet-I cloned into miniCTX-1-P*lac*, Tet^R	This study
pRL662-*gfp2*	pRL662 (39) derivative expressing GFP2, $Gent^R$	Erh-Min Lai lab
pKNG101	Gene replacement suicide vector, R6K *ori*, *sacB*, Str^R	(4)
pUT18	Bacterial-two-hybrid vector encoding the T18 fragment of *Bordetella pertussis* adenylate cyclase to construct N-terminal fusions, Amp^R	(13)
pUT18C	Bacterial-two-hybrid vector encoding the T18 fragment of *Bordetella pertussis* adenylate cyclase to construct C-terminal fusions, Amp^R	(13)
pKNT25	Bacterial-two-hybrid vector encoding the T25 fragment of *Bordetella pertussis* adenylate cyclase to construct N-terminal fusions, Kan^R	(13)
pUT18C_TagB1^{PP}	T18 fragment of *Bordetella pertussis* adenylate cyclase C-terminally fused to TagB1^{PP}, Amp^R	This study
pUT18_TagB1^{PP}	T18 fragment of *Bordetella pertussis* adenylate cyclase N-terminally fused to TagB1^{PP}, Amp^R	This study

pKNT25_TssE1^{PP}	T25 fragment of *Bordetella pertussis* adenylate cyclase N-terminally fused to TssE1^{PP}, Kan^{R}	This study
pKNT25_TssF1^{PP}	T25 fragment of *Bordetella pertussis* adenylate cyclase N-terminally fused to TssF1^{PP}, Kan^{R}	This study
pKNT25_TssG1^{PP}	T25 fragment of *Bordetella pertussis* adenylate cyclase N-terminally fused to TssG1^{PP}, Kan^{R}	This study
pKNT25_TssK1^{PP}	T25 fragment of *Bordetella pertussis* adenylate cyclase N-terminally fused to TssK1^{PP}, Kan^{R}	This study
pKNT25_Hcp1^{PP}	T25 fragment of *Bordetella pertussis* adenylate cyclase N-terminally fused to Hcp1^{PP}, Kan^{R}	This study
pKNT25_VgrG1^{PP}	T25 fragment of *Bordetella pertussis* adenylate cyclase N-terminally fused to VgrG1^{PP}, Kan^{R}	This study
pKNT25_TssA1^{PP}	T25 fragment of *Bordetella pertussis* adenylate cyclase N-terminally fused to TssA1^{PP}, Kan^{R}	This study
pKNT25_TssB1^{PP}	T25 fragment of *Bordetella pertussis* adenylate cyclase N-terminally fused to TssB1^{PP}, Kan^{R}	This study
pKNT25_TssJ1^{PP}	T25 fragment of *Bordetella pertussis* adenylate cyclase N-terminally fused to TssJ1^{PP} lacking the peptide leader sequence, Kan^{R}	This study
pKNT25_TssL1^{PP}	T25 fragment of *Bordetella pertussis* adenylate cyclase N-terminally fused to cytosolic sequence of TssL1^{PP}, Kan^{R}	This study
pKNG101-*tssA1*^{PP}	PCR fragment containing the regions upstream and downstream *tssA1*^{PP} cloned in pKNG101 for double recombination; when inserted into the chromosome and the plasmid cured, the strain is a *tssA1*^{PP} mutant, Str^{R}	(26)
pKNG101-*tagB1*^{PP}	PCR fragment containing the regions upstream and downstream *tagB1*^{PP} cloned in pKNG101; when inserted into the chromosome and the plasmid cured, the strain is a *tagB1*^{PP} mutant, Str^{R}	This study
pKNG101-*tagB1*^{PP}*compl*	PCR fragment containing *tagB1*^{PP} and its upstream and downstream regions cloned in pKNG101; when inserted into the chromosome of a *tagB1*^{PP} mutant and the plasmid cured, the mutant strain is complemented for *tagB1*^{PP}, Str^{R}	This study
pKNG101-*hcp1*^{PP}	PCR fragment containing the regions upstream and downstream *hcp1*^{PP} cloned in pKNG101; when inserted into the chromosome and the plasmid cured, the strain is a *hcp1*^{PP} mutant, Str^{R}	This study
pKNG101-*tssB1*^{PP}*::sfgfp*	PCR fragment containing the 3´ region of *tssB1*^{PP} fused to *sfgfp* and the downstream region of the same gene cloned in pKNG101; when inserted into the chromosome and the plasmid cured, the strain expresses TssB1^{PP} C-terminally fused to sfGFP, Str^{R}	This study
pKNG101-*tssB1*^{PP}*::mScarlet-I*	PCR fragment containing the 3´ region of *tssB1*^{PP} fused to *mScarlet-I* and the downstream region of the same gene cloned in pKNG101, when inserted into the chromosome and the plasmid cured, the strain expresses TssB1^{PP} C-terminally fused to mScarlet-I, Str^{R}	This study
pKNG101-*tssA1*^{PP}*::sfgfp*	PCR fragment containing the 5´ region of *tssA1*^{PP}	This study

	fused to *sfgfp* and the upstream region of the same gene cloned in pKNG101, when inserted into the chromosome and the plasmid cured, the strain expresses TssA1^{PP} N-terminally fused to sfGFP, Str^{R}	
pKNG101-*tagB1*^{PP}*::sfgfp*	PCR fragment containing the 3´ region of *tagB1*^{PP} fused to *sfgfp* and the downstream region of the same gene cloned in pKNG101, when inserted into the chromosome and the plasmid cured, the strain expresses TagB1^{PP} C-terminally fused to sfGFP, Str^{R}	This study
pKNG101-*tssB1*^{PA}*::sfgfp*	PCR fragment containing the 3´ region of *tssB1*^{PA} fused to *sfgfp* and the downstream region of the same gene cloned in pKNG101, when inserted into the chromosome and the plasmid cured, the strain expresses TssB1^{PA} C-terminally fused to sfGFP, Str^{R}	This study
pKNG101-*tssB2*^{PA}*::sfgfp*	PCR fragment containing the 3´ region of *tssB2*^{PA} fused to *sfgfp* and the downstream region of the same gene cloned in pKNG101, when inserted into the chromosome and the plasmid cured, the strain expresses TssB2^{PA} C-terminally fused to sfGFP, Str^{R}	This study
pKNG101-*tssB3*^{PA}*::sfgfp*	PCR fragment containing the 3´ region of *tssB3*^{PA} fused to *sfgfp* and the downstream region of the same gene cloned in pKNG101, when inserted into the chromosome and the plasmid cured, the strain expresses TssB3^{PA} C-terminally fused to sfGFP, Str^{R}	This study
pKNG101-*tagJ1*^{PA}*::sfgfp*	PCR fragment containing the 3´ region of *tagJ1*^{PA} fused to *sfgfp* and the downstream region of the same gene cloned in pKNG101, when inserted into the chromosome and the plasmid cured, the strain expresses TagJ1^{PA} C-terminally fused to sfGFP, Str^{R}	This study
pKNG101-*tagB1*^{PP}*::(StrepII)*$_{2}$	PCR fragment containing the 3´ region of *tagB1*^{PP} fused to a twin StrepII tag and the downstream region of the same gene cloned in pKNG101, when inserted into the chromosome and the plasmid cured, the strain expresses TagB1^{PP} C-terminally fused to two consecutive StrepII tags, Str^{R}	This study
pRK600	Helper plasmid, ColE1 *ori*, *mob*RK2, *tra*RK2, Cam^{R}	(40)

Table S3. Oligonucleotide primers used in this study. The “Brief description” column provides basic information on the primer design (restriction enzyme(s) used for cloning, encoded protein or gene replaced by antibiotic resistance cassette, forward or reverse orientation of the primer (F or R)). Primers marked with TE (for “tag exchange”) were used for replacing an existing affinity tag with an alternative one, primers marked with UP, DOWN or MED were used for construction of mutant strains, primers marked with T18 or T25 were used for construction of plasmids used in bacterial-two-hybrid experiments, and primers marked with CTX were used for the construction of miniCTX vector derivatives for insertion of genes into the *Pseudomonas* genome. Superscripts “*PP*” and “*PA*” indicate *P. putida* and *P. aeruginosa* components (genes or proteins), respectively; these abbreviations are solely used in this table for brevity.

Number	Brief description	Sequence (5´-3´)
P1	NdeI.Hcp1^{PP}.F	ggccggcatatgatgttgttaatggagagtttc
P2	BamHI.Hcp1^{PP}.R	tacgcaggatccggttcggtacgtgcttag
P3	NdeI.TagB1^{PP}.F	aattaacatatggacgatgcaccaatgac
P4	XhoI.TagB1^{PP}StrepII.R	ttaattctcgagctacttttcgaactgcgggtggctccacagtcctt cgatcgtcagc
P5	EcoRI.TssA1^{PP}.F	gcgctgaattcgcctgaaattctcaatgacctc
P6	HindIII.TssA1^{PP}.R	tggttaagcttgacgttcgtctcaaggctt
P7	NdeI.TagJ1^{PA}.F	gcttacatatggccgacccttcattcgc
P8	XbaI.TagJ1^{PA}StrepII.R	aaaaaagatctacttttcgaactgcgggtggctccaggcgtccg tcggctcgaag
P9	EcoRI.TssA1^{PA}.F	atcgagaattccgtgctggatgtacccg
P10	HindIII.TssA1^{PA}.R	tggccaagcttccgctaggcgttactcg
P11	TE.TssA1^{PP}V5.F	accgaggagagggttagggataggcttacccatggtatatctcc ttcttaaagttaaac
P12	TE.TssA1^{PP}V5.R	cctaaccctctcctcggtctcgattctacgcctgaaattctcaatg acctctcgctg
P13	TE.TagB1^{PP}V5.F	accgaggagagggttagggataggcttacccagtccttcgatc gtcagcagcaggcgctg
P14	TE.TagB1^{PP}V5.R	cctaaccctctcctcggtctcgattctacgtagctcgagtctggta aagaaaccgctgctgc
P15	TE.TssA1^{PA}V5.F	accgaggagagggttagggataggcttacccatggtatatctcc ttcttaaagttaaac
P16	TE.TssA1^{PA}V5.R	cctaaccctctcctcggtctcgattctacgctggatgtacccgtttt gctggctg
P17	TE.TagJ1^{PA}V5.F	accgaggagagggttagggataggcttaccggcgtccgtcgg ctcgaagtccagttcac
P18	TE.TagJ1^{PA}V5.R	cctaaccctctcctcggtctcgattctacgtagatctcaattggata tcggccggccacgcg
P19	XbaI.tagB1^{PP}UP.F	ggttaatctagagctgcgccggtgtt
P20	tagB1^{PP}UP.R	ttacagtccatcgtccatgtgcctgct
P21	tagB1^{PP}DOWN.F	atggacgatggactgtaacgcgcagcg
P22	BamHI.tagB1^{PP}DOWN.R	ttatcaggatccgtcgccggcctgtt
P23	XbaI.tagB1^{PP}-gfpUP.F	ggttcctctagactggtcgaactctacgaacgt
P24	tagB1^{PP}-gfpUP.R	tgctgctgccagtccttcgatcgtcag
P25	tagB1^{PP}-gfpMED.F	gaaggactggcagcagcaggaggagga
P26	tagB1^{PP}-gfpMED.R	cgctgcgcgtcattacttatacagctc
P27	tagB1^{PP}-gfpDOWN.F	ataagtaatgacgcgcagcgcccggcacc
P28	BamHI.tagB1^{PP}-gfpDOWN.R	aattaaggatccttcacggccgaattccttgc

P29	XbaI.tssA1^{PP}-gfpUP.F	ggttcctctagaacaaggactggatcgaagtgc
P30	tssA1^{PP}-gfpUP.R	tttacgcatagaacactctggacatgg
P31	tssA1^{PP}-gfpMED.F	gagtgttctatgcgtaaaggcgaagaa
P32	tssA1^{PP}-gfpMED.R	tcctcctcctgctgctgccttatacagctcgtccat
P33	tssA1^{PP}-gfpDOWN.F	gcagcagcaggaggaggacctgaaattctcaatgac
P34	BamHI.tssA1^{PP}-gfpDOWN.R	aattaaggatcctgctgtctgcatcgccttg
P35	XbaI.tssB1^{PP}-gfpUP.F	ggttcctctagattcgcagcgaattgctcg
P36	tssB1^{PP}-gfpUP.R	tgctgctgcggcgcgatcgtcgctgga
P37	tssB1^{PP}-gfpMED.F	gatcgcgccgcagcagcaggaggagga
P38	tssB1^{PP}-gfpMED.R	ggctcctgcttacttgtacagctcgtc
P39	tssB1^{PP}-gfpDOWN.F	tacaagtaagcaggagcccgaacatgac
P40	BamHI.tssB1^{PP}-gfpDOWN.R	aattaaggatccagtcaccgatcaacatgccg
P41	tssB1^{PP}-mScMED.F	gatcgcgccgcagcagcaggaggaggaatggttagcaaagg cgaggc
P42	tssB1^{PP}-mScMED.R	ggctcctgcttaggatcccttatacagttcgtccatg
P43	tssB1^{PP}-mScDOWN.F	ggatcctaagcaggagcccgaacatgaccgacaagc
P44	SacI.tssB1^{PP}-mScCTX.F	aattatcccgggtaacaggaggaattaaccatgattcgcagcga attgctcg
P45	XmaI.tssB1^{PP}-mScCTX.R	aattccgagctctgcttaggatcccttatacagttcgtc
P46	XbaI.tssB2^{PA}-gfpUP.F	ggttcctctagattatcgcaggctgtgccacc
P47	tssB2^{PA}-gfpUP.R	tgctgctgcgtcctgggagggggcggc
P48	tssB2^{PA}-gfpMED.F	tcccaggacgcagcagcaggaggagga
P49	tssB2^{PA}-gfpMED.R	ctagggggtggcttacttatacagctcgtccataccg
P50	tssB2^{PA}-gfpDOWN.F	aagtaagccaccccctagccaaggaag
P51	BamHI.tssB2^{PA}-gfpDOWN.R	aattaaggatcccgctggagacgtattgcatc
P52	XbaI.tssB3^{PA}-gfpUP.F	ggttcctctagagggactgcgatgatcagggcatcc
P53	tssB3^{PA}-gfpUP.R	tgctgctgcggccggctggtcggccgg
P54	tssB3^{PA}-gfpMED.F	cagccggccgcagcagcaggaggagga
P55	tssB3^{PA}-gfpMED.R	ccgggaagagggttacttatacagctcgtccataccg
P56	tssB3^{PA}-gfpDOWN.F	aagtaaccctcttcccggagaagccgc
P57	BamHI.tssB3^{PA}-gfpDOWN.R	aattaaggatccagcaggccgatgtcctgc
P58	XbaI.tagJ1^{PA}-gfpUP.F	aaaaatctagagccgatggtacagacctactccaccg
P59	tagJ1^{PA}-gfpUP.R	tgctgctgcggcgtccgtcggctcgaagtcc
P60	tagJ1^{PA}-gfpMED.F	ccgacggacgccgcagcagcaggaggagga
P61	tagJ1^{PA}-gfpMED.R	cgcaggtcgaacttacttatacagctcgtccataccg
P62	tagJ1^{PA}-gfpDOWN.F	tataagtaagttcgacctgcgtgaactggacttcgagc
P63	BamHI.tagJ1^{PA}-gfpDOWN.R	tataaggatccgccggtctccaggtccaggttg
P64	XbaI.T18-tagB1^{PP}.F	gcggtctagaggacgatgcaccaatgacaca
P65	BamHI.T18-tagB1^{PP}.R	gcgtggatccttacagtccttcgatcgtcag
P66	XbaI.tagB1^{PP}-T18.F	gcgatctagagatggacgatgcaccaatgac
P67	BamHI.tagB1^{PP}-T18.R	atatggatcctccagtccttcgatcgtcag
P68	XbaI.tssE1^{PP}-T25.F	gcgcgtctagagagtaaccaggcccgcctgct
P69	BamHI.tssE1^{PP}-T25.R	atatggatcctcgttgaggttctgcactttg
P70	XbaI.tssF1^{PP}-T25.F	gcgcgtctagagctcgacgaactgctgccctа
P71	BamHI.tssF1^{PP}-T25.R	atatggatcctcgaccaggggctgatc
P72	XbaI.tssG1^{PP}-T25.F	gcgcgtctagaggccagcacgcaccggcgatca
P73	BamHI.tssG1^{PP}-T25.R	atatggatcctccaaggcgaccccatcgtaag
P74	XbaI.tssK1^{PP}-T25.F	gcgcgtctagagagcaagcagagccgggtgat
P75	BamHI.tssK1^{PP}-T25.R	atatggatcctctttgagcaccgccatcagtt
P76	XbaI.hcp1^{PP}-T25.F	cgcgctctagagttgttaatggagagtttcacaatg
P77	BamHI.hcp1^{PP}-T25.R	atatggatcctcggcaaacactttgttcgcgg
P78	XbaI.vgrG1^{PP}-T25.F	gcgcgtctagagctaactgacgttctttcc

P79	BamHI.vgrG1^{PP}-T25.R	atatggatcctcgtcgttgatccgcactgc
P80	XbaI.tssA1^{PP}-T25.F	gcgcgtctagagcctgaaattctcaatgacc
P81	BamHI.tssA1^{PP}-T25.R	atatggatcctcggtgcccagcacatcgcg
P82	XbaI.tssB1^{PP}-T25.F	gcgctctagagattcgcagcgaattgc
P83	BamHI.tssB1^{PP}-T25.R	atatggatcctcggcgcgatcgtcgctgga
P84	XbaI.tssJ1^{PP}-T25.F	gcgcgtctagagggggtcaccgaccgtatcg
P85	BamHI.tssJ1^{PP}-T25.R	atatggatcctctttgacgctatccactaaag
P86	XbaI.tssL1c^{PP}-T25.F	gcgcgtctagagaccgaagccgtactgcaac
P87	BamHI.tssL1c^{PP}-T25.R	atatggatcctccgacacctggtcgggcagt
P88	XbaI.tssB1^{PA}-gfpUP.F	gggaagcactaccagcagtcagaagttcat
P89	tssB1^{PA}-gfpUP.R	cctgctgctgccgcctgcgg
P90	tssB1^{PA}-gfpMED.F	gcagcagcaggaggaggaatgcgtaaaggcgaagaa
P91	tssB1^{PA}-gfpMED.R	atcctctcattacttatacagctcgtccat
P92	tssB1^{PA}-gfpDOWN.F	taatgagaggattccagcatggccgaattgag
P93	BamHI.tssB1^{PA}-gfpDOWN.R	gtagtagtcgccgaccaggcagcc
P94	XbaI.tagB1^{PP}-StrepII2UP.F	ggttcctctagattggcgaatacctgcaaggc
P95	tagB1^{PP}-StrepII2UP.R	gtggctccacttttcgaactgcgggtggctccacagtccttcgat cgtcagca
P96	tagB1^{PP}-StrepII2DOWN.F	ttcgaaaagtggagccacccgcagttcgaaaagtagctgacga tcgaaggactg
P97	BamHItagB1^{PP}-StrepII2DOWN.R	aattaaggatcctgcggtttgcttgtcggtc
P98	XbaI.hcp1^{PP}UP.F	taatctagactgcgcgaccgctac
P99	hcp1^{PP}UP.R	gcgcggaattcgtaaagggttgctcatgc
P100	hcp1^{PP}DOWN.F	gcagaattcgcacgtaccgaaccat
P101	BamHIhcp1^{PP}DOWN.R	aatggatccttgcggttcgctctg

LEGENDS FOR SUPPLEMENTARY INFORMATION VIDEOS

Video S1. *In vivo* imaging of *P. putida rpoN* TssB1-sfGFP. The full cycle of T6SS assembly over approximately 120 seconds is shown for a representative sheath. This video was generated using a fluorescence microscopy time-lapse recording (total time 140 seconds) of *P. putida rpoN* expressing TssB1-sfGFP from the native *tssB1* locus. Images were acquired every 2 s and the video is presented at 7 frames/s.

Video S2. *In vivo* imaging of *P. putida rpoN* TagB1-sfGFP. TagB1 forms transient foci which accumulate and abruptly disappear over time. Disappearance of foci is not due to photobleaching as new TagB1-sfGFP foci appear in the same or neighboring cells. This video was generated using a fluorescence microscopy time-lapse recording (total time 120 seconds) of *P. putida rpoN* expressing TagB1-sfGFP from the native *tagB1* locus. Images were acquired every 2 s and the video is presented at 7 frames/s.

LEGENDS FOR SUPPLEMENTARY INFORMATION DATASETS

Dataset S1. Overview of the results originating from an *in silico* analysis of the structural components of 100 T6SS clusters. The T6SS clusters included in the analysis are organized by phylogenetic group based on the classification by Boyer et al. (20) (groups 1-5 are shown in different tabs, with phylogenetic subgroups 4A and 4B shown separately). 20 T6SS clusters per phylogenetic group were analyzed and a hyperlink to the SecReT6 website (19) is given for each cluster; strains appearing in multiple phylogenetic groups have multiple T6SSs. The T6SS cluster that is best characterized in each phylogenetic group (highlighted in grey) was set as the reference cluster for the analysis (distinct clusters from *P. aeruginosa*, *E. coli*, *P. putida* and *A. tumefaciens* were selected as references). Cells containing identity and coverage metrics are color coded according to their bit score as provided by the NCBI blastp search tool (21). Bit score is an indicator of sequence similarity that is independent of query sequence length and database size and that is normalized based on the raw pairwise alignment score; red cells indicate the highest scores (score > 200), i.e. most similar alignments, followed by magenta (score = 80-200), green (score = 50-80), blue (score = 40-50) and black cells (score < 40). White cells indicate proteins that cannot be found in a given T6SS cluster. In some cases, more than one analogue of a specific T6SS core component is found in a given cluster; for example there are two TssC analogues in all T6SS clusters of phylogenetic group 5, several T6SS clusters have multiple VgrG or Hcp components, and in approximately 2% of cases there are two analogues of some components of the membrane complex or the baseplate. These additional components are not shown for simplicity, and the protein that is most similar to that of the reference genome is included in the spreadsheet.

Dataset S2. *P. putida* TagB1-(StrepII)$_2$ specifically co-purifies with TssA1. Proteins eluted from Strep-Tactin Sepharose resin incubated with *P. putida rpoN* or *P. putida rpoN tagB1-(StrepII)$_2$* whole-cell lysates, as identified and quantified by mass spectrometry. The file contains the output of a volcano plot analysis, performed in Perseus version 1.5.8.5 (9); proteins identified in the TagB1-(StrepII)$_2$ eluate were compared to those identified in the control eluate (untagged *P. putida* TagB1). Proteins with a positive “difference” value are enriched in the TagB1-(StrepII)$_2$ condition compared to the control, whilst proteins with a negative “difference” value are enriched in the control compared to the TagB1-(StrepII)$_2$ condition. Proteins with significantly different abundances in the two conditions are marked

"YES" in the "Significant" column. Log_2 LFQ intensity values, in technical triplicate, are given for each protein sample in each condition.

SUPPLEMENTARY INFORMATION REFERENCES

1. K.-H. Choi, A. Kumar, H. P. Schweizer, A 10-min method for preparation of highly electrocompetent *Pseudomonas aeruginosa* cells: application for DNA fragment transfer between chromosomes and plasmid transformation. *J. Microbiol. Meth.* **64**, 391-397 (2005).

2. M. I. Ramos-Gonzalez, E. Duque, J. L. Ramos, Conjugational transfer of recombinant DNA in cultures and in soils: host range of *Pseudomonas putida TOL* plasmids. *Appl. Environ. Microb.* **57**, 3020-3027 (1991).

3. P. Vasseur, I. Vallet-Gely, C. Soscia, S. Genin, A. Filloux, The *pel* genes of the *Pseudomonas aeruginosa* PAK strain are involved at early and late stages of biofilm formation. *Microbiology* **151**, 985-997 (2005).

4. K. Kaniga, I. Delor, G. R. Cornelis, A wide-host-range suicide vector for improving reverse genetics in Gram-negative bacteria: inactivation of the *blaA* gene of *Yersinia enterocolitica*. *Gene* **109**, 137-141 (1991).

5. T. T. Hoang, A. J. Kutchma, A. Becher, H. P. Schweizer, Integration-proficient plasmids for *Pseudomonas aeruginosa*: site-specific integration and use for engineering of reporter and expression strains. *Plasmid* **43**, 59-72 (2000).

6. S. Tyanova, T. Temu, J. Cox, The MaxQuant computational platform for mass spectrometry-based shotgun proteomics. *Nat. Protoc.* **11**, 2301-2319 (2016).

7. J. Cox *et al.*, Andromeda: a peptide search engine integrated into the MaxQuant environment. *J. Proteome Res.* **10**, 1794-1805 (2011).

8. J. Cox *et al.*, Accurate proteome-wide label-free quantification by delayed normalization and maximal peptide ratio extraction, termed MaxLFQ. *Mol. Cell. Proteom.* **13**, 2513-2526 (2014).

9. S. Tyanova *et al.*, The Perseus computational platform for comprehensive analysis of (prote)omics data. *Nat. Methods* **13**, 731-740 (2016).

10. A. Hachani, N. S. Lossi, A. Filloux, A visual assay to monitor T6SS-mediated bacterial competition. *J. Vis. Exp.*, e50103-e50103 (2013).

11. L.-S. Ma, A. Hachani, J.-S. Lin, A. Filloux, E.-M. Lai, *Agrobacterium tumefaciens* deploys a superfamily of type VI secretion DNase effectors as weapons for interbacterial competition *in planta*. *Cell Host Microbe* **16**, 94-104 (2014).

12. A. Hachani *et al.*, Type VI Secretion System in *Pseudomonas aeruginosa* secretion and multimerization of VgrG proteins. *J. Biol. Chem.* **286**, 12317-12327 (2011).

13. G. Karimova, J. Pidoux, A. Ullmann, D. Ladant, A bacterial two-hybrid system based on a reconstituted signal transduction pathway. *Proc. Natl. Acad. Sci. U. S. A.* **95**, 5752-5756 (1998).

14. J. D. Pedelacq, S. Cabantous, T. Tran, T. C. Terwilliger, G. S. Waldo, Engineering and characterization of a superfolder green fluorescent protein. *Nat. Biotechnol.* **24**, 79-88 (2006).

15. D. S. Bindels *et al.*, mScarlet: a bright monomeric red fluorescent protein for cellular imaging. *Nat. Methods* **14**, 53-56 (2017).

16. J. Schindelin *et al.*, Fiji: an open-source platform for biological-image analysis. *Nat. Methods* **9**, 676-682 (2012).

17. A. Ducret, E. M. Quardokus, Y. V. Brun, MicrobeJ, a tool for high throughput bacterial cell detection and quantitative analysis. *Nat. Microbiol.* **1**, 16077 (2016).

18. P. Thevenaz, U. E. Ruttimann, M. Unser, A pyramid approach to subpixel registration based on intensity. *IEEE Trans. Image Proc.* **7**, 27-41 (1998).

19. J. Li *et al.*, SecReT6: a web-based resource for type VI secretion systems found in bacteria. *Environ. Microbiol.* **17**, 2196-2202 (2015).

20. F. Boyer, G. Fichant, J. Berthod, Y. Vandenbrouck, I. Attree, Dissecting the bacterial type VI secretion system by a genome wide *in silico* analysis: what can be learned from available microbial genomic resources? *BMC Genomics* **10**, 104-104 (2009).

21. S. F. Altschul, W. Gish, W. Miller, E. W. Myers, D. J. Lipman, Basic local alignment search tool. *J. Mol. Biol.* **215**, 403-410 (1990).

22. I. Letunic, T. Doerks, P. Bork, SMART: recent updates, new developments and status in 2015. *Nucleic Acids Res.* **43**, D257-D260 (2015).

23. J. R. Otero-Asman, S. Wettstadt, P. Bernal, M. A. Llamas, Diversity of extracytoplasmic function sigma (s^{EFC}) factor-dependent signaling in *Pseudomonas*. *Mol. Microbiol.* **112**, 356-373 (2019).

24. D. T. Jones, W. R. Taylor, J. M. Thornton, The rapid generation of mutation data matrices from protein sequences. *Comput. Appl. Biosci.* **8**, 275-282 (1992).

25. I. Letunic, P. Bork, Interactive Tree Of Life (iTOL) v4: recent updates and new developments. *Nucleic Acids Re.s* **47**, W256-W259 (2019).

26. P. Bernal, L. P. Allsopp, A. Filloux, M. A. Llamas, The *Pseudomonas putida* T6SS is a plant warden against phytopathogens. *ISME J.* **11**, 972-987 (2017).

27. S. R. Dix *et al.*, Structural insights into the function of type VI secretion system TssA subunits. *Nat. Commun.* **9**, 4765-4765 (2018).

28. J. Schneider *et al.*, Diverse roles of TssA-like proteins in the assembly of bacterial type VI secretion systems. *EMBO J.* **38**, e100825-e100825 (2019).

29. D. Hanahan, in DNA cloning: a Practical Approach, D. M. Glover, Ed. (IRL Press, McLean, Virginia, 1985), vol. 1, pp. 109.

30. M. Herrero, V. de Lorenzo, K. N. Timmis, Transposon vectors containing non-antibiotic resistance selection markers for cloning and stable chromosomal insertion of foreign genes in Gram-negative bacteria. *J. Bacteriol.* **172**, 6557-6567 (1990).

31. H. W. Boyer, D. Roulland-Dussoix, A complementation analysis of the restriction and modification of DNA in *Escherichia coli*. *J. Mol. Biol.* **41**, 459-472 (1969).

32. F. W. Studier, B. A. Moffatt, Use of bacteriophage T7 RNA polymerase to direct selective high-level expression of cloned genes. *J. Mol. Biol.* **189**, 113-130 (1986).

33. M. Espinosa-Urgel, A. Salido, J.-L. Ramos, Genetic analysis of functions involved in adhesion of *Pseudomonas putida* to seeds. *J. Bacteriol.* **182**, 2363-2369 (2000).

34. T. Köhler, S. Harayama, J. L. Ramos, K. N. Timmis, Involvement of *Pseudomonas putida* RpoN sigma factor in regulation of various metabolic functions. *J. Bacteriol.* **171**, 4326-4333 (1989).

35. L. P. Allsopp *et al.*, RsmA and AmrZ orchestrate the assembly of all three type VI secretion systems in *Pseudomonas aeruginosa*. *Proc. Natl. Acad. Sci. U. S. A.* **114**, 7707-7712 (2017).

36. L.-S. Ma, J.-S. Lin, E.-M. Lai, An IcmF family protein, $ImpL_M$, is an integral inner membrane protein interacting with $ImpK_L$, and its Walker A motif is required for type VI secretion system-mediated Hcp secretion in *Agrobacterium tumefaciens*. *J. Bacteriol.* **191**, 4316-4329 (2009).

37. M. Jovanovic *et al.*, Regulation of the co-evolved HrpR and HrpS AAA+ proteins required for *Pseudomonas syringae* pathogenicity. *Nat. Commun.* **2**, 177-177 (2011).

38. I. Pérez-Martínez, L. Rodriguez-Moreno, I. Matas, C. Ramos, Strain selection and improvement of gene transfer for genetic manipulation of *Pseudomonas savastanoi* isolated from olive knots. *Res. Microbiol.* **158**, 60-69 (2007).

39. A. C. Vergunst *et al.*, VirB/D4-dependent protein translocation from *Agrobacterium* into plant cells. *Science* **290**, 979-982 (2000).

40. B. Kessler, V. de Lorenzo, K. N. Timmis, A general system to integrate *lacZ* fusions into the chromosomes of Gram-negative eubacteria: regulation of the *Pm* promoter of the *TOL* plasmid studied with all controlling elements in monocopy. *Mol. Gen. Genet.* **233**, 293-301 (1992).
